# Fabrication and Characterization of Eco-Friendly Thin Films as Potential Optical Absorbers for Efficient Multi-Functional Opto-(Electronic) and Solar Cell Applications

**DOI:** 10.3390/ma16093475

**Published:** 2023-04-29

**Authors:** Mohamed H. El-Newehy, Ahmed M. El-Mahalawy, Badr M. Thamer, Meera Moydeen Abdul Hameed

**Affiliations:** 1 Department of Chemistry, College of Science, King Saud University, Riyadh 11451, Saudi Arabia; bthamer@ksu.edu.sa (B.M.T.);; 2 Department of Chemistry, Faculty of Science, Tanta University, Tanta 31527, Egypt; 3 Thin Films Laboratory, Physics Department, Faculty of Science, Suez Canal University, Ismailia 41522, Egypt; ahmed_elsayed@science.suez.edu.eg

**Keywords:** dyed chitosan thin films, laser cut-off filter, nonlinear optical parameters, heterojunction photosensors, solar energy conversion

## Abstract

The necessity for reliable and efficient multifunctional optical and optoelectronic devices is always calling for the exploration of new fertile materials for this purpose. This study leverages the exploitation of dyed environmentally friendly biopolymeric thin films as a potential optical absorber in the development of multifunctional opto-(electronic) and solar cell applications. Uniform, stable thin films of dyed chitosan were prepared using a spin-coating approach. The molecular interactivity between the chitosan matrix and all the additive organic dyes was evaluated using FTIR measurements. The color variations were assessed using chromaticity (CIE) measurements. The optical properties of films were inspected using the measured UV-vis-NIR transmission and reflection spectra. The values of the energy gap and Urbach energy as well as the electronic parameters and nonlinear optical parameters of films were estimated. The prepared films were exploited for laser shielding as an attenuated laser cut-off material. In addition, the performance of the prepared thin films as an absorbing organic layer with silicon in an organic/inorganic heterojunction architecture for photosensing and solar energy conversion applicability was studied. The current-voltage relation under dark and illumination declared the suitability of this architecture in terms of responsivity and specific detectivity values for efficient light sensing applications. The suitability of such films for solar cell fabrications is due to some dyed films achieving open-circuit voltage and short-circuit current values, where Saf-dyed films achieved the highest *V_oc_* (302 mV) while MV-dyed films achieved the highest *J_sc_* (0.005 mA/cm^2^). Finally, based on all the obtained characterization results, the engineered natural cost-effective dyed films are considered potential active materials for a wide range of optical and optoelectronic applications.

## 1. Introduction

Despite the dependence of global economic growth on rapid industrial development supported by continuous experimental and laboratory research results, global public awareness has ignored the negative effects and hazards on the environment and human health resulting from the overreliance on fuel-based manufacturing processes of synthetic plastics. In this context, over the last few years, many countries have resorted to the exploitation of eco-friendly materials and sustainability concepts for supporting economic growth along with social requirements, in addition to tackling climate change and environmental protection. This can be achieved by replacing harmful toxic chemical materials for manufacture with eco-friendly natural materials for industry [1,2,3].

On the scale of the field of optoelectronics and optical devices, there is no doubt that lead, cadmium, arsenic, and selenium are among the most common and widely used elements in the solar cell industry, achieving high efficiencies and outstanding performance, in addition to their use in many optical devices, such as optical lenses and filters. It is worth noting that these materials are highly toxic, and this is what the Silicon Valley Toxics Coalition’s stated: “There are potential negative environmental and health impacts from PV modules throughout their life cycles, ranging from raw-materials extraction and procurement impacts, toxic and hazardous materials used in manufacturing, and the disposal and recycling of modules at the end of their useful lives” [1,2,3]. Additionally, on the first of July 2006, European legislation panned marketing any electronic device containing more than 0.1% weight in a unit of Cd [4].

Accordingly, a natural material such as chitosan was chosen, and due to its weak absorption in the visible spectral range, the chitosan was dyed with several colored organic dyes, which have maximum absorption at different wavelengths along the visible electromagnetic spectrum. Chitosan is one of the most important natural hydrophilic biopolymers that can be obtained from the N-deacetylate chitin that exists in abundance (15–40%) in crustacean shell biomasses. According to the deacetylation degree and the source of chitin, the polymorphic form of chitosan is determined; α-chitosan is extracted from arthropods’ exoskeletons (α-chitin), while β-chitosan is extracted from squid pens (β-chitin) [4]. The molecular structure of chitosan is a combination between randomly distributed β-linked N-acetyl-D-glucosamine and D-glucosamine in a linear polysaccharide structure [5]. The power of chitosan as a natural superior polymer arises from its abundance and its environmental friendliness in addition to the ease of exploitation in many biomedical and pharmaceutical applications, owing to its high bio-adhesivity, promising antibacterial activity, efficient wound healing characteristics, and outstanding biocompatibility [6]. The physicochemical properties of chitosan can be tailored easily by tuning the molar mass, polydispersity index, deacetylation degree, and pattern, which, in turn, can yield further functionalities [4]. Chitosan can be prepared easily in various forms, i.e., powder, gel, membrane, nanofibers, resins, and nanoparticles [5,7]. In the environmental application field, this sustainable material has been exploited in desalination and water purification from heavy metals, dyes, fluoride, herbicides, radionuclides, and pesticides in addition to soil treatments (i.e., biostimulation and degradation of xenobiotic compounds) [8,9]. Furthermore, chitosan has been used for food preservation, tissue engineering, biodiesel generation [10], and fuel cell engineering [11].

On the other side, chitosan has been exploited in many electronic and optoelectronic applications, such as photodiodes [12], thermal sensors [13], Schottky barrier modifier [13,14], resistive random-access memory (RRAM) [15,16], light-emitting diodes [17], humidity sensors [18,19], modulators [20], and optical waveguides, where the lower-molecular-weight chitosan achieved lower loss than that of the higher molecular weight [21]. Additionally, chitosan possessed unique photochromic features [22] and corrosion inhibition [23]. Furthermore, it has been used for fabricating piezoelectric human health sensors [24] and utilized as an electrolyte in dye-synthesized solar cells (DSSCs) [25].

It is heartening to mention that, in addition to all the aforementioned properties of chitosan, it intrinsically has high interaction activity due to the existence of several functional groups such as hydroxyl and amino groups, which give it the feasibility to form composites and blends [26]. This opened the way toward a wider scale of efficient applications based on chitosan. Several previous studies succeeded in tailoring the linear/nonlinear optical properties of different polymers, such as PVA [5,6,7,8] and PMMA [9,10], utilizing different dyes for laser-limiting applications. Therefore, the optoelectronic properties of chitosan can be tuned via the dyeing process, where the spectral absorption range can be shifted and photoprotective features can be altered. This may be suitable for controllable optical sensing applications and building windows’ material engineering with higher purified indoor media properties [27].

Therefore, we adopted the idea of dyeing chitosan films to improve their optical absorption properties and study the effect of the dyeing process on the color and optical constants of films, in addition to studying the optical shielding properties of the prepared films as an initial step for their adoption as eco-friendly materials for windows, sterile places, and food preservation. In addition, we investigated the effect of the dyeing process on the photosensing properties of the engineered films in a hybrid heterojunction configuration. The dyeing process was carried out using eleven different organic dyes, ranging from violet to red, to cover the entire visible spectrum.

## 2. Materials and Methods

### 2.1. Materials

Chitosan powder, Cs, of molecular weight 300-350 kDa, purchased from Spectrum Chemical Co., New Brunswick, NJ, USA, was utilized as the main material for the present study. All the utilized organic dyes were purchased from Sigma Aldrich, St. Louis, MO, USA. The molecular structure as well as the molecular weight of each organic dye are illustrated in Table S1 (Supporting Information).

### 2.2. Preparation of Thin Films

**Figure 1:**
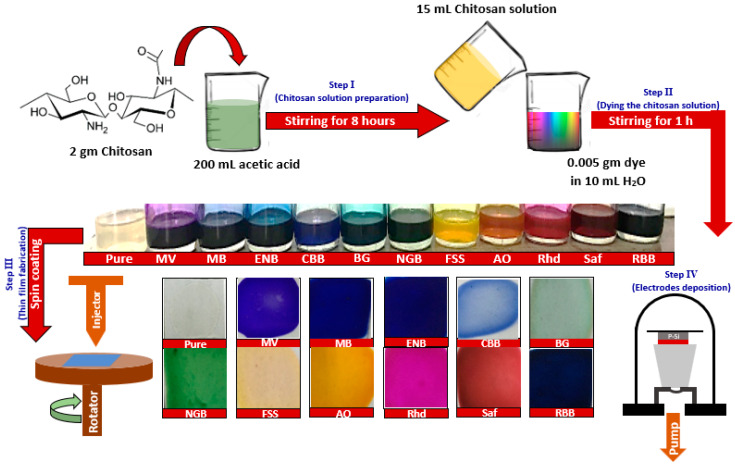
Scheme of fabrication of dyed chitosan thin films and sensors.

The preparation approach that was employed to fabricate the dyed thin films is shown in Figure 1. Firstly, 2 g of chitosan powder was dissolved in 200 mL of (2%) acetic acid through magnetic stirring at room temperature for 8 h. The obtained solution was left for 1 h to confirm that the solution was clear and agglomerate-free. Then, the organic dye solutions were prepared by dissolving 0.005 g of each dye in 10 mL of deionized water at room temperature for 2 h. Secondly, the dyed chitosan solutions were fabricated by adding 15 mL of the pure chitosan solution (virgin) to the dissolved dye solution under magnetic stirring for another 1 h. The estimated viscosity coefficient values of the prepared solutions using BROOKFIELD AMETEK DV1 Digital Viscometer under 100 rpm and 27 °C are listed in Table S2 (Supporting Information). The virgin and dyed thin films were deposited using the spin-coating technique at 3000 rpm on glass, quartz, and p-type silicon substrates. The spinning time for each film was 70 s under quasi-static spinning mode at room temperature. The deposited films were left in the open air until the solvent was vaporized. Finally, the deposited polymeric films on the pre-etched p-type <100> silicon were placed in an Edwards coating unit for depositing aluminum and silver as bottom ohmic and upper Schottky electrodes, respectively, at base pressure 3 × 10^−8^ bar for implementation of metal/polymer/semiconductor (MPS) heterojunction, as shown in Figure S1 (Supporting Information) for light sensing applications.

### 2.3. Characterization Techniques

The molecular structures of the pure and dyed chitosan films were investigated using Bruker ALPHA FTIR spectrophotometer in a spectral range (400–4000) cm^−1^. Furthermore, optical transmission and reflection spectra of the fabricated thin films in the UV-visible-NIR were assessed using a Jasco V-770 double-beam spectrophotometer in a range of (190–2500) nm. The influence of the dyeing of pure chitosan thin films on its emission spectrum was inspected using a Shimadzu RF-5301 PC spectrofluorophotometer under a 350 nm excitation source. The shielding characteristics of the designed films for visible light were tested under a green laser diode (532 nm). The colorimetric parameters of the engineered films were identified using UltraScan Pro. (HunterLab, Reston, VA, USA). All color measurements were taken under the conditions of the standard illuminant D65 and 10° observers, as described by the Commission Internationale de L’éclairage (CIE L* a* b* standard).

Eventually, the light sensitivity of the designed metal/polymer/semiconductor (MPS) architectural junctions employing silver as a rectifier metal electrode, virgin and dyed chitosan as a polymeric film, and p-type silicon as a semiconductor was evaluated under halogen light of irradiance 100 mW/cm^2^ under forward and reverse bias of driving voltage in the range ±3 volts using Keithley 6516B electrometer.

## 3. Results

### 3.1. FTIR Molecular Structure Characterization

**Figure 2:**
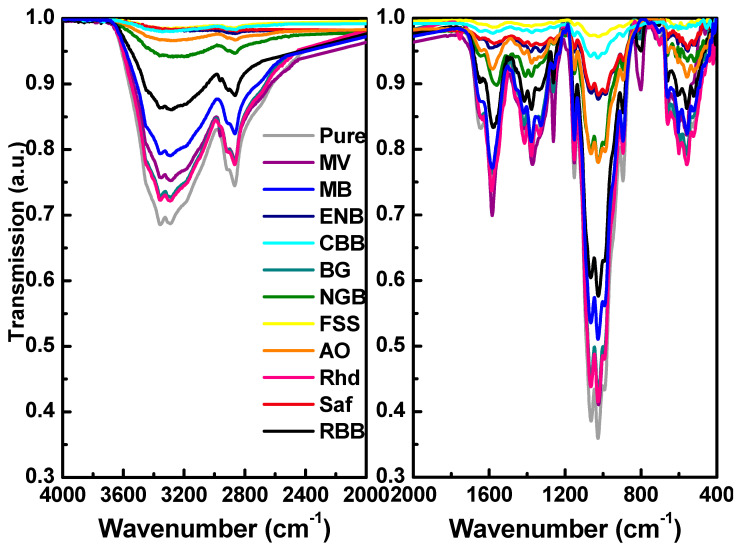
FTIR spectra of the pure and dyed chitosan thin films.

The molecular vibrations and interactions between the chitosan matrix and incorporated organic dyes were probed using the collected FITR spectra shown in Figure 2. Superficially, the shape of the IR spectra of all films may be relatively similar. The IR peak positions and their corresponding molecular vibration identification are illustrated in Table 1. The detected IR peaks of pure Cs are well-defined, with a high degree of deacetylation (DD %) of about 81.25% [28,29]. The observed IR band in the range of (3356–3293) cm^−1^ is attributed to two overlapped stretching vibrations of -OH and -NH [30,31]. This band was perturbed (hypsochromic and bathochromic) owing to the intermolecular hydrogen bonding between the incorporated organic dyes and the chitosan molecules [29,30]. The IR peaks at 2917 and 2867 cm^−1^ are ascribed to symmetric and asymmetric stretching vibrations of C-H, respectively, of polysaccharides. The C=O of primary and secondary amides stretch at 1646 and 1583 cm^−1^, respectively. In addition, the NH bending and stretching vibrations of amide I overlapped at 1583 cm^−1^ [28,30]. The features of the C-H chains’ side bending and NH_2_ wagging appear in the observed IR peak at 1419 cm^−1^. The detected IR peak at 1376 cm^−1^ is assigned to the C=O bending mode and C-H methyl group torsion. The amide III C-N stretching vibration is observed at 1326 cm^−1^, while the asymmetric stretching of the C-O-C bridge is observed at 1151 cm^−1^ [28,30,31]. Furthermore, the glucosamine C-O stretching and glucose C-O bending vibrations are detected at 1063 cm^−1^, while the C-N stretching β-glycosidic band peak is located at 895 cm^−1^ [28,30,31]. The out-of-plane bending of N-H and C-O vibrations are detected at 707 and 559 cm^−1^, respectively. The strong IR peak at 1029 cm^−1^ is relevant to the free amino group at C_2_ of glucosamine, where these protonated free amino groups act as active sites for attracting ionic compounds, leading to better miscibility between the chitosan and incorporated organic dyes [29,30]. The hypsochromic and bathochromic shifts in wavenumber values confirm the good interactivity between molecular structures of pure and dyed chitosan films. It should be mentioned that MV, Rhd, and CBB dyes achieved the highest value of IR absorption at 3356, 3292, and 1598 cm^−1^, indicating the strongest interaction between NH_3_^+^ groups of chitosan and the dye molecules [28].

### 3.2. Spectrophotometric Optical Characterizations

#### 3.2.1. Transmission and Reflection Spectra

**Figure 3:**
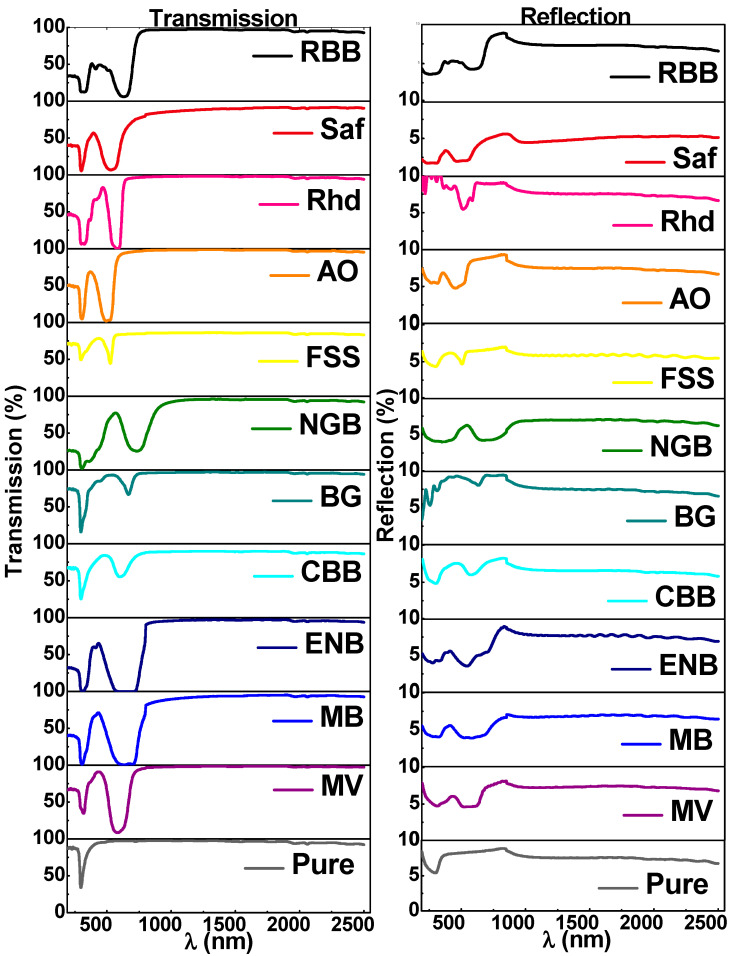
UV-vis-NIR transmission and reflection spectra of the pure and dyed chitosan thin films.

To examine the optical properties of the prepared films, the transmission, *T*(*λ*), and reflection, *R*(*λ*), of the prepared thin films dyed with different dyes were measured in the spectrum range extending from UV to NIR, as shown in Figure 3. As for the *T*(*λ*) profile, all the prepared films exhibit the same behavior in the infrared region (800–2500) nm since all the prepared films achieve high values of transparency (90 < *T*(*λ*) <100%). However, in contrast, in the visible and UV spectral bands, the pigmentation process resulted in many noticeable changes, the first of which is that the fundamental absorption edge of pure Cs thin films at 299 nm was related to the light absorption by amino acids and was red shifted to different values regarding the impregnating dye, but the NGB dye achieved the highest blue shift in the fundamental absorption edge at 665 nm [32,33]. The neglected observance of interference fringes in the spectral profile of transmittance is conclusive evidence for the uniformity of the films’ thicknesses [34]. In addition, the tendency of transmittance, in some cases (e.g., MB, ENB, NGB, and Rhd), to vanish suggests the fabricated dyed films can be used for eco-friendly optical shielding materials. According to the transmission spectra, the deposited films’ thicknesses were determined using a point-wise unconstrained minimization approach (PUMA) and retrieved to be 393 ± 13 nm for all films. Table S3 (Supporting Information) lists the film thickness of each film. On the other side, the *R*(*λ*) profiles are significantly different for the dyed and virgin Cs thin films, but on the whole, all the films exhibit low reflection values, especially in the NIR region *R*(*λ*) ≤ 10%. In the NIR, for some of the samples, T and R sum up to more than 100%. This may be due to employing an aluminum mirror as a reference during reflection measurements, which yield appreciably varied reflectance values.

Interference fringes in transmission and reflection spectra arise in the case of a relatively high film thickness, and they are a good indicator of the uniformity of film thickness. Mathematical treatments can be resolved using the dependence of absorption coefficient and refractive index on T, R, k, or depending on the Swanepoel algorithm. Owing to the absence of interference fringes of some films rather than others, we used the following equations for calculating α and n to unify the mathematical calculation approach.

#### 3.2.2. Absorption Coefficient, Energy Gap, and Urbach Energy

The spectral functionalities of the engineered thin films depend mainly on their absorption features; thereby, the absorption coefficient, *α* (*λ*), of the fabricated thin films was calculated as follows [35]:

(1) $$\alpha =\frac{1}{t}\ln\left[\left(\frac{{\left(1-R\right)}^{2}}{2T}\right)+\sqrt{\left(\left(\frac{{\left(1-R\right)}^{4}}{4{\left(T\right)}^{2}}\right)+{\left(R\right)}^{2}\right)}\right],$$

where *t* is the thin film’s thickness.

**Figure 4:**
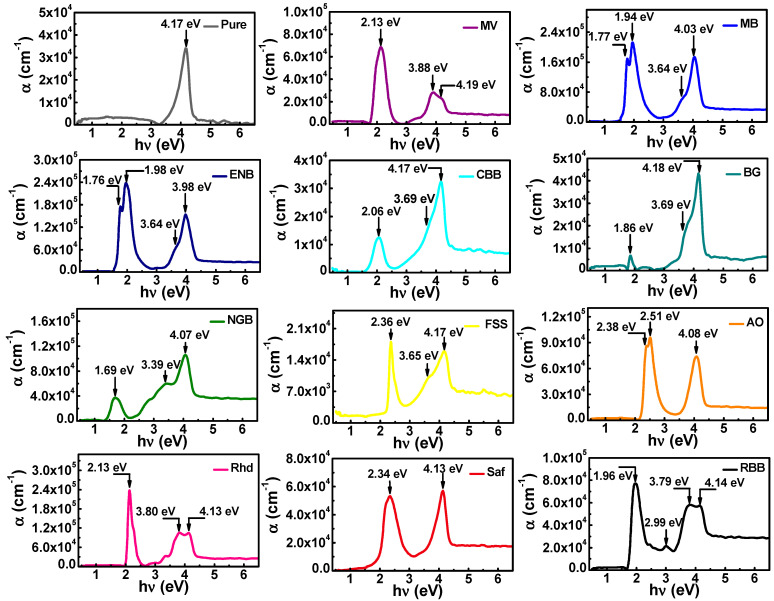
Photon energy dependence of absorption coefficient of the pure and dyed chitosan thin films.

Figure 4 reveals the photon energy reliance of the absorption coefficient in all the prepared thin films. It can be noticed that pure chitosan possesses a single UV absorption peak (Soret band) at 4.17 eV, which is attributed to *π*-π^*^ electronic transition of the chromophoric C=O [36,37]. Upon dyeing chitosan with different dyes, the values of absorption were enhanced exceeding (*α* > 10^5^ cm^−1^ for MB, ENB, NGB, and Rhd), and new sharp absorption bands developed in the visible spectral region. The developed absorption peaks in the visible region are attributed to the electronic transitions between the non-boding molecular orbital and the anti-bonding molecular orbitals (*n*→π^*^) of the dyes [38,39]. The astonishing visible and UV absorption features of the dyed thin films increase their potentiality for light harvesting utility in solar cells and light sensing applications.

Conspicuously, a strong molecular interaction was achieved between the chitosan molecules and added dyes, which rationalizes the variation in the shape of the characteristic Soret band of pure chitosan [36,40]. This change in the Soret band’s shape is represented either in the splitting of the absorption peak, as observed in the case of MV, Rhd, and RBB, or the appearance of a shoulder attached to it, as observed in the case of MB, ENB, CBB, BG, NGB, and FSS. The splitting of the Soret band is an indicator of a strong interaction, which can be interpreted via Davydov splitting phenomena, where the coupling of the vibronic state would result in a split in the excitation states when the Cs and (MV, Rhd, and RBB) molecules interact. This splitting depends on the inert molecular separation as well as transition dipole moment orientation [41]. On the other side, the appearance of a shoulder in the case of MB, ENB, CBB, BG, NGB, and FSS may be either owing to the absorption of free charge carriers or activation of the (*n*→π^*^) electronic transitions of chitosan [36,37,38,39,40,41]. Regarding the solar spectrum that extends from UV to IR regions, the fabricated films’ absorption features covered the visible region extending from the violet to red region, in addition to the UV absorption features of chitosan. Hence, referring to the solar spectrum, the intense wavelength (500 nm) may provide enough energy to be absorbed by many prepared dyed films, the closest of which is dyed by CBB, FSS, AO, Rhd, and Saf. Meanwhile, chitosan-dyed films with MV, MB, ENB, AO, Saf, and RBB achieve the highest absorption coefficient in the spectral regions of the solar spectrum, which may yield a better photo-energy conversion performance.

For unveiling the impact of the dyeing of the electronic structure of the designed Cs thin films, the Tauc relation was employed for estimating the values of the inter-band energy gap, *E_g_*, as follows [34,35,39]:

(2) $$\alpha =\frac{B}{h\nu }{\left(h\nu -E_{g}\right)}^{m},$$

where *hν* is photon energy, while *m* is a constant that indicates the type of electronic transition near the band edge (*m* = ½ and 2 for direct and indirect, respectively).

Figure S2 (Supporting Information) depicts the Tauc plot for a direct energy gap estimation of the prepared films. Extrapolating the linear region of the (*αhν*)^2^ data to null, the corresponding energy is related to the energy gap. The obtained *E_g_*_1_ of the virgin Cs thin film is about 3.82 eV, which is smaller than that reported previously in a search of the literature, such as (~4.23 eV [14]), (~5.31 eV [37]), (~5.2 [42]), and theoretical HOMO-LUMO gap (5.876 eV) but higher than others (~3.36 eV [14]) and (~3.4 eV [26]). This discrepancy may arise from the difference in the molecular weight of chitosan in each study. As observed in Table 2, after dyeing, the fundamental energy gap decreased, where NGB achieved the lowest value (2.88 eV). Meanwhile, the dyeing induced the formation of sub-band energy levels that enhance the photoexcitation process via the electronic inter-band transitions, leading to another *E_g_*_2_, as listed in Table 2.

For a more detailed inspection of the influence of the dyeing process on the chitosan’s electronic structure and how its structural disorder and compositional irregularities affect its electronic band structure, Urbach energy, *E_u_*, which represents the tail state width of the fabricated thin films, was calculated as follows [35]:

(3) $$\alpha =\alpha_{o}\exp\left(\frac{h\nu }{E_{u}}\right),$$

Regarding the slope of the relation between ln*α* versus *hν* that is sketched in Figure S3 (Supporting Information), the values of *E_u_* were estimated. Accordingly, the virgin Cs thin films achieved *E_u_* of about 243 meV, which is smaller than those determined in the literature (2450 meV [26]), (930 meV [37]), and (1250 meV [42]). The difference between the estimated Urbach energy in the present study and other reports may reflect the high quality, lower structural imperfections, and polycrystallinity of the fabricated chitosan films. In addition, the small value of *E_u_* is a good factor in the case of using chitosan in solar cells to reduce the process of the recombination of charge carriers due in those tail states. As can be observed in Table 2, the dyeing of chitosan achieved obvious alternations in tail energy, where NGB, FSS, CBB, and RBB achieved the highest tailing states’ energy, declaring the highest perturbation of the electronic structure, resulting in denser localized states in the gap in these cases.

#### 3.2.3. Dispersion Behavior and Dispersive Parameters

To assess the suitability of the designed films for optical applications, such as optical communication waveguides and optical windows, it is worth discussing the interactivity in the incident electromagnetic waves and the designed films in terms of wave dispersion, which depends mainly on the microstructure of the films. Consequently, the linear refractive index, *n*, was calculated using the following relation [34,35,38,39,43]:

(4) $$n=\sqrt{\left(\frac{4R}{{\left(1-R\right)}^{2}}-{\left(\frac{\alpha \lambda }{4\pi }\right)}^{2}\right)}+\left(\frac{1+R}{1-R}\right),$$

where *λ* is the wavelength of the incident electromagnetic wave. The dispersion behavior of the calculated refractive indices of the prepared films that are revealed in Figure S4 (Supporting Information) comprises two behaviors; one is the anomalous dispersion in the spectral range *λ* < 850 nm, while the other is the normal dispersion behavior (2500 > *λ* > 850) nm. The normal dispersion behavior was analyzed following the assumptions of the Wemple–DiDomenico model of the singularity in the oscillator responsible for the dispersion, where the refractive index is interrelated to the incident photon energy as follows [34,35,38,39,43]:

(5) $$n^{2}-1=\frac{E_{o}E_{d}}{\left[E_{o}^{2}-{\left(h\nu \right)}^{2}\right]},$$

where *E_d_* and *E_o_* are the dispersion and oscillator energies, respectively.

**Figure 5:**
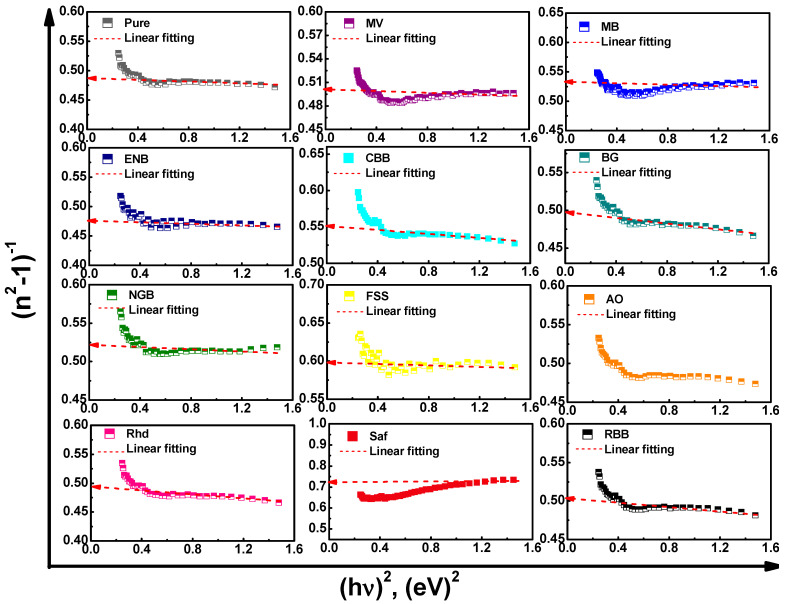
(*n*^2^ − 1)^−1^ versus (*hν*)^2^ single oscillator model plots of the pure and dyed chitosan thin films.

Thereby, the relation between (*n*^2^ − 1)^−1^ versus (*hν*)^2^ that is plotted in Figure 5 can be exploited for estimating the dispersive parameters. The estimated values of *E_d_* and *E_o_* of the virgin Cs films are about 19.3 and 9.36 eV, respectively, which are higher than those in previous studies [26,37]. Meanwhile, the addition of colorful dyes resulted in significant variations in the *E_d_* and *E_o_* of the virgin thin films, as shown in Table 2. Furthermore, the static refractive index, *n_o_*, and infinite dielectric constant, *ε*_∞_, can be deduced using the following relation [34,35,38,39,43]:

(6) $$\varepsilon_{\infty }=n_{o}^{2}=1+\frac{E_{d}}{E_{o}},$$

It should be noted that the static refractive index of pure Cs is larger than that obtained in previous work, which may be due to the difference in molecular weight [26,32,33,37,44]. The tailored behavior of the refractive index of Cs thin films upon dyeing, as observed in Table 2, opens the way for the effective exploitation of these materials as a sustainable source for designing and implementing controllable non-lossy optical fibers and anti-reflective coatings.

Otherwise, the whole dielectric behavior of such films consists of two contributions; one arises from the lattice, *ε_L_*, and the other is due to the free charge carriers of the number per unit effective mass, *N*/m*, as follows [34,35,38,39,43]:

(7) $$n^{2}=\varepsilon_{L}-\frac{e^{2}}{4\pi^{2}\varepsilon_{o}c^{2}}\left(\frac{N}{m^{*}}\right)\lambda^{2},$$

Without regarding the extinction coefficient values in the transparent region, the slope of intercept of the relation between *λ*^2^ and *n*^2^ that is drawn in Figure S5 (Supporting Information) would give rise to the values of *ε_L_* and *N*/m*, as observed in Table 2. The dyed chitosan thin films from ENB, BG, AO, and Rhd recorded the highest free charge carriers’ density per effective mass.

Regarding the superposition between the hypothesis of the single oscillator model and classical free electron model, some featured electronic parameters of the engineered films can be derived, including plasma frequency, the plasma energy of the valance electrons, Fermi energy, and Penn energy (i.e., average energy gap), as follows [35,45]:

(8) $$\omega_{P}=e\sqrt{\left(\frac{N}{\varepsilon_{o}\varepsilon_{L}m^{*}}\right)},$$

(9) $$\psi =\hbar \omega_{P},$$

(10) $$E_{P}=\frac{\psi }{\sqrt{\left(\varepsilon_{\infty }-1\right)}},$$

(11) $$E_{F}=0.2948\times {\left(\psi \right)}^{\frac{4}{3}},$$

where *ω_P_*, *ψ*, *E_P_*, and *E_F_* stand for plasma frequency, valance electron plasma energy, Penn energy, and Fermi energy, respectively. The calculated values of the mentioned parameters are listed in Table 2 and showed a significant variation upon using different organic dyes. This confirms that the incorporation of the organic dyes into the chitosan structure effectively tuned its electronic structure for tailored optical and electrical properties.

#### 3.2.4. Nonlinear Optical Parameters

The optical nonlinearity arises from the linearity deviation in the relation between the probed electric field and the resulting polarization. The deviation in polarization away from the linearity can be enhanced by the incorporation of many more donor and acceptor function groups and by improving the molecular π-conjugation length. The non-centrosymmetric structure with a high conjugation of the incorporated dyes allows for the transportation of charge from donor to acceptor units, which, in turn, can result in higher nonlinearity [46]. The versatility of the designed thin films for nonlinear applications as modulators, optical filters, waveguides, and optical storing devices can be deduced from the calculated nonlinear parameters, such as third-order nonlinear optical susceptibility, *χ*^(3)^, and nonlinear refractive index, *n*_2_. These parameters can be extracted using the Miller rule and Wemple–DiDomenico model for photon energy trends to vanish as follows [34,35,46]:

(12) $$\chi^{\left(3\right)}=A{\left[\chi^{\left(1\right)}\right]}^{4}=A{\left[\frac{E_{d}E_{o}}{4\pi E_{o}^{2}}\right]}^{4},$$

(13) $$n_{2}=\frac{12\pi \chi^{\left(3\right)}}{n_{o}},$$

where *χ*^(1)^ is the first-order linear optical susceptibility and *A* is a constant (~1.7 × 10^−10^ esu). The thin films of dyed Cs by ENB achieved the highest nonlinear parameters but, in contrast, the films of chitosan dyed by Saf achieved the lowest nonlinear optical parameters.

### 3.3. Photoluminescence and Color Chromaticity Characterizations

**Figure 6:**
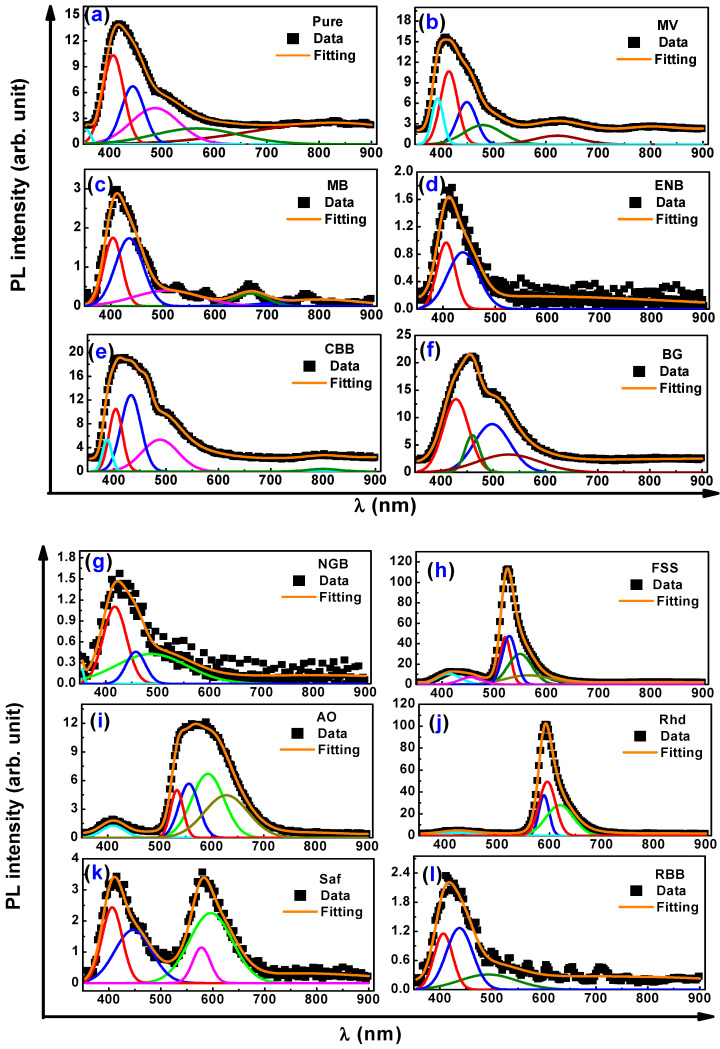
Resolved photoluminescence spectra of pure (**a**–**f**) and dyed chitosan (**g**–**l**) thin films at excitation wavelength 350 nm.

Certainly, the shape of the photoluminescence spectral curve and its intensity is a clear indication of the extent of the interaction between the chitosan matrices and the added dyes. The intermolecular interaction between the incorporated conjugated dyes and the main chains of chitosan would enhance the π-electron delocalization, which, in turn, alters the PL intensity. Figure 6a–i emphasize the resolved PL spectra of the virgin and dyed chitosan films with sub-band vibrational molecular contributions to the whole spectrum [47]. Obviously, the virgin Cs film displays a violet emission peak at 417 nm. Interestingly, upon incorporating the different dyes into the chitosan matrix, the PL declared new strong emission peaks in addition to the native emission peak of chitosan, as in the case of FSS (524 nm), AO (570 nm), Rhd (592 nm), and Saf (582 nm), as envisioned in Figure 6h–k, respectively. Meanwhile, the PL emission spectra of chitosan were enhanced because of the emission of the impregnating dye, such as BG (456 nm), CBB (418 nm), and MV (408 nm), showing overlapped convoluted spectra. Otherwise, the PL emission was quenched upon the incorporation of other dyes, such as MB, ENB, NGB, and RBB. The PL deterioration may be owing to either the complexation between the hydroxyl groups of chitosan and metal ions, as in the case of RBB and NGB, which change the electron–hole recombination annihilation or electrostatic interaction between the protonated amine groups of chitosan (polycationic character) and the incorporated dyes bearing anionic character, such as MB and ENB.

Indeed, the coordinate shift in emission color is significantly observed in the 1931 CIE gamut diagram shown in Figure S6 (Supporting Information). In addition, the CIE XY coordinates of each color were calculated, as listed in Table 3. To qualify the effectiveness of designed films to be employed as filters, the color strength shown in Figure S7 (Supporting Information) and the estimated color parameters provided in Table 3 were evaluated. The lowest lightness value, L*, was observed under the RBB dyeing of chitosan, owing to its black color, while the highest change in color, dE*, was observed when Rhd dye was incorporated into the chitosan matrix. The increment in the a* value from −0.81 for virgin chitosan to 17.07 and −9.39 upon dying by Rhd and NGB illustrates the reinforcement of reddish and greenish tones, respectively. Meanwhile, the elevation of b* values from −1.34 for virgin chitosan film to 16.97 and −12.17 upon dying by AO and MV illustrates the reinforcement of yellowish and bluish tones, respectively. These features increase the potentiality of the engineered films for spectral selective filtering and shielding applications.

### 3.4. Laser Cut-Off Filter Performances

**Figure 7:**
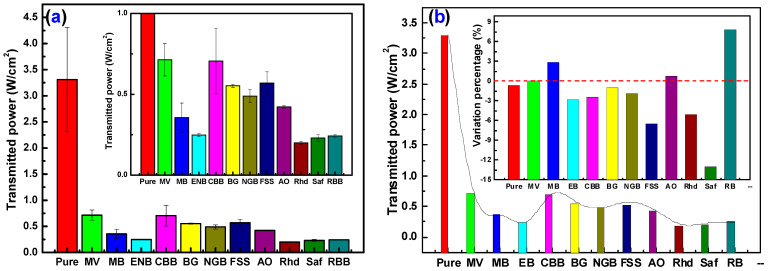
(**a**) Impregnating dye dependence of laser cut-off filter performance of pure and dyed chitosan films and (**b**) aging effect on the cut-off performance.

Confirming the ability of the prepared dyed films to be used as an effective filter and cut-off for visible light, the shielding behavior of the dyed chitosan films for a green laser of incident intensity (7.46 W/cm^2^) was tested. Figure 7a represents the transmitted light intensity from each film. It can be noticed that the virgin chitosan film exhibited low attenuation for the incident green light with transmittance (*I_T_*/*I_o_*~44.4%), while Rhd-dyed chitosan films showed the lowest transmittance (*I_T_*/*I_o_*~2.68%). These results agree with the discussed colorimetric and optical measurement results. Evaluating the stability and activity of the fabricated films as visible light filters, the experiment was repeated after keeping the fabricating films under ambient dark conditions for 6 months, as shown in Figure 7b. The pure and dyed films exhibited high stability with very low variations in the transmittance after 6 months. As observed in the inset in Figure 7b, the highest transmittance enhancement was observed in the case of the RBB-dyed chitosan film by 7.82%, while the highest transmittance deterioration was observed in the Saf-dyed chitosan film by −13%.

### 3.5. Photosensing Performance

**Figure 8:**
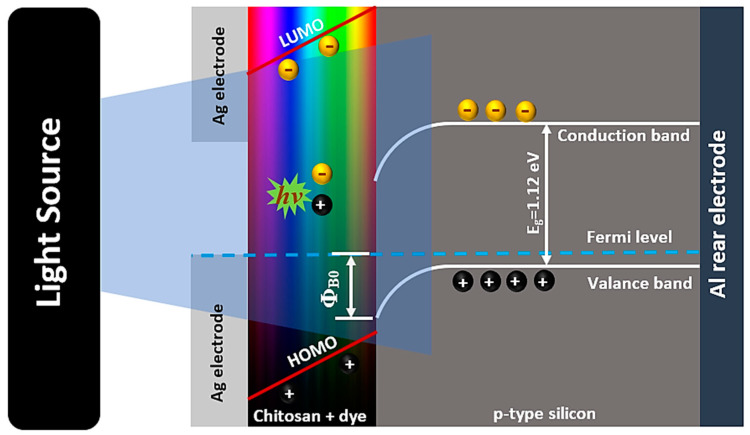
Schematic diagram and band diagram of the designed heterojunction device.

**Figure 9:**
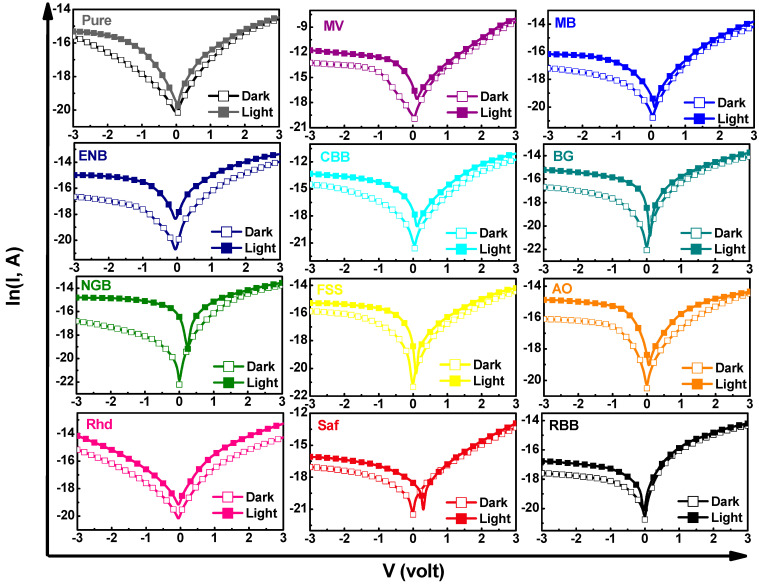
Semi-logarithmic current–voltage relation of the pure and dyed chitosan thin films integrated with p-Si heterojunction under dark and light conditions.

In a completely different way, to unravel the photosensing capability of the engineered films, the prepared films were used to remediate the spectral deficiencies in silicon to manufacture a photosensor in the form of a metal/insulator/semiconductor (MIS) heterojunction. The spectral selectivity of the designed photosensor of the architecture shown in Figure 8 can be tailored according to the absorption features of the deposited dyed chitosan on a silicon substrate, which may enhance the light-harvesting leverage [38,48]. Since the incorporated polymeric interlayer can be considered as a Schottky barrier height modifier of MS Schottky junction, the current–voltage (*I*-*V*) measurements of the designed photosensors were recorded under dark and illumination conditions. The photosensing capability of these fabricated devices was evaluated from the semilogarithmic current–voltage relations, as shown in Figure 9. It is worth noting that all the fabricated devices exhibit a linear current–voltage dependence relation in the forward low-bias region, but in high-biasing regions, the high parasitic series resistance, *R_s_*, makes the relation deviate from linearity. Meanwhile, under reverse biasing, the reverse current increases monotonically as the reverse bias voltage increases, but in some devices (i.e., MV, MB, ENB, CBB, BG, NGB, FSS, AO, Saf, and RBB), the reverse current shows a quasi-voltage independence behavior, revealing the efficient passivation of the Si surface by the spin-coated dyed chitosan films.

Otherwise, all the fabricated devices show a rectification behavior, where the highest rectification ratio (*RR*~137) under dark conditions is observed in the case of the Ag/Cs-MV/p-Si device, as shown in Figure S8a (Supporting Information). The difference in the obtained low values of RR compared to the previously reported values of Co/chitosan/p-Si [13] may be ascribed to the difference between the work function of the utilized electrodes [38,49]. Following is the thermionic emission model for estimating the microelectronic parameters of the designed non-ideal junctions (*n* > 1) of series resistance, *R_s_*, where the current–voltage–temperature (*I*-*V*-*T*) is represented as follows [34,38,39,46]:

(14) $$I=I_{s}exp\left(\frac{q\left(V-IR_{s}\right)}{nk_{B}T}\right)\left[1-\exp\left(\frac{-q\left(V-IR_{s}\right)}{k_{B}T}\right)\right],$$

(15) $$I_{s}=AA^{*}T^{2}exp\left(\frac{-q\Phi_{B0}}{k_{B}T}\right),$$

where *k_B_* is the Boltzmann constant, *A* is the active area of the implemented device, A* is the Richardson constant, *I_s_* is the reverse saturation current, and Φ*_B0_* is the zero-bias barrier height. As observed in Figure S8b (Supporting Information), all the fabricated devices possess nonideal Schottky diode behavior, since the lowest *n*-value (~2.16) was observed for Ag/Cs-NGB/p-Si. The nonideality of the fabricated devices may be rationalized because of the formation of a native oxide layer between Si and the spin-coated chitosan film that develops sufficient series resistance in addition to the hypothetical consequences of the existence of variable heights in the Schottky barrier height (SBH) [12,13,14,38,39,46,50]. In addition, the developed interface states density between the chitosan and p-Si as well as the image forces and tunneling mechanism of charges may contribute to the nonideality of the present devices [12,13,14,38,39,46,50]. Likewise, the dark reverse saturation current and barrier height were estimated and plotted, as shown in Figure S8c,d (Supporting Information), respectively. The lowest achieved reverse current (*I_s_* = 0.23 nA) was obtained also in the case of the Ag/Cs-NGB/p-Si device. However, the highest barrier height (Φ*_B_*_0_ = 0.87 eV) was observed in the case of Ag/Cs-NGB/p-Si and Ag/Cs-CBB/p-Si devices. It is worth noting that the insertion of chitosan films between the Ag electrode and the p-type silicon increased the Ag/p-Si barrier significantly by 90 meV for Ag/Cs-NGB/p-Si and 20 meV for Ag/Cs-Rhd/p-Si. The interlayered chitosan film between Ag and Si manipulated the interaction between them by reducing the metal’s work function and electron affinity of the semiconductor, which resulted in a higher barrier height. It is well known that the controllability of the barrier height depends on the characteristics of the inserted organic film between the metal/semiconductor interface, such as its HOMO and LUMO energetic position, and their position relative to semiconductor electron affinity and electrode work function. The obtained values of Φ*_B0_* of all the fabricated devices are higher than those previously recorded for Au/Cs/n-Si (~0.73 eV [23]) and Co/Cs/p-Si (~0.68 eV [13]) but lower than those estimated for Ag/Cs/n-Si (~0.94 eV [22] and Ag/Cs/n-Si (~0.88 eV [12]). The values of shunt resistance, *R_sh_*, and series resistance, *R_s_*, of the fabricated devices were estimated from the junction’s resistance, *R_j_*, and plotted, as shown in Figure S8e,f (Supporting Information), respectively. On the scale of shunt resistance, Ag/Cs-MV/p-Si and Ag/Cs-BG/p-Si devices achieved the lowest values, 25.7 MΩ and 9.9 MΩ, respectively. However, regarding the series resistance, the lowest values were observed for Ag/Cs-MV/p-Si and Ag/Cs-CBB/p-Si as 36.5 kΩ and 32.3 kΩ, respectively.

It should be considered that the superficial nano-roughness defects, native oxide layer, and lattice mismatch between Si and the chitosan layer are responsible for initiating interface states, which act as recombination trapping centers. These states increase the series resistance of the fabricated heterojunction in addition to the resistances of the undepleted region of silicon and the contact electrode. The interface state density, *N_ss_*, of each device was calculated and plotted as a function of its energetic position relative to the valance band maxima, *E_ss_-E_v_* [38,39], as shown in Figure S9 (Supporting Information). It can be noticed that all the devices exhibit a decayed interfacial state density profile as the value of *E_ss_-E_v_* increases. Firstly, the lowest density of these states was observed for the Ag/Cs-MV/p-Si device ~3.56 × 10^−13^ eV^−1^cm^−2^, and secondly, the energetic position of these states is 0.5-*E_v_* eV, while the energetic position of these states in the other devices shifts to a higher energetic position ~0.66-*E_v_* eV. Thereby, it can be concluded that the low density of interface trapping states and the short scale of the distribution relative to valance band maxima in the A/Cs-MV/p-Si heterojunction limits its role in the generated e-h pair recombination.

Furthermore, under halogen illumination, the produced reverse current of each device was increased significantly, as observed in Figure 9. For discussing the insight aspects of charge photogeneration and collection in the designed heterojunction in the light of the suggested band diagram, Figure 8 was used for confirmation. The incident photons of energy, *hυ*, can be absorbed by the organic chitosan layer generating electron–hole (e–h) pairs at the interfacial heterojunction depletion region, which can be split and swept with the help of the junction’s built-in voltage. The split holes can be drifted towards the p-Si side overcoming the developed barrier height, while electrons can be drifted towards the chitosan layer collected by the electrodes under the conditions of the reverse bias voltage generating the photocurrent. The interfacial trapping states between the silicon and chitosan organic layer can induce recombination of these charges, impeding the photogeneration process. It should be mentioned that the highly penetrated incident photons may result in generating deep e–h pairs, which would diffuse toward the junction, contributing to the produced photocurrent by slow diffusing charges. Notably, the Ag/Cs-MV/p-Si device achieved the highest photocurrent, *J_photo_* = *J_illumination_* − *J_dark_*, (~0.29 mA/cm^2^), as observed in Figure S10 (Supporting Information). The efficiency of separation and sweeping of photogenerated e–h pairs were elucidated on the appearance of open-circuit current *I_sc_* [*I* = *I_sc_*]_V=0_ and open-circuit voltage *V_oc_* [*V* = *V_oc_*]_I=0_ due to the photovoltaic effect in all the devices, except in the case of pure Rhd-dyed chitosan film devices, as shown in Figure S11 (Supporting Information). The maximum observed value of *V_oc_* was about 246 mV for the Ag/Cs-NGB/p-Si device, while the maximum *J_sc_* was about 0.005 mA/cm^2^ for the Ag/Cs-MV/p-Si device. This increases the effectiveness of the prepared devices for exploitation in the field of self-powered photodetection applications and solar cell applications.

**Figure 10:**
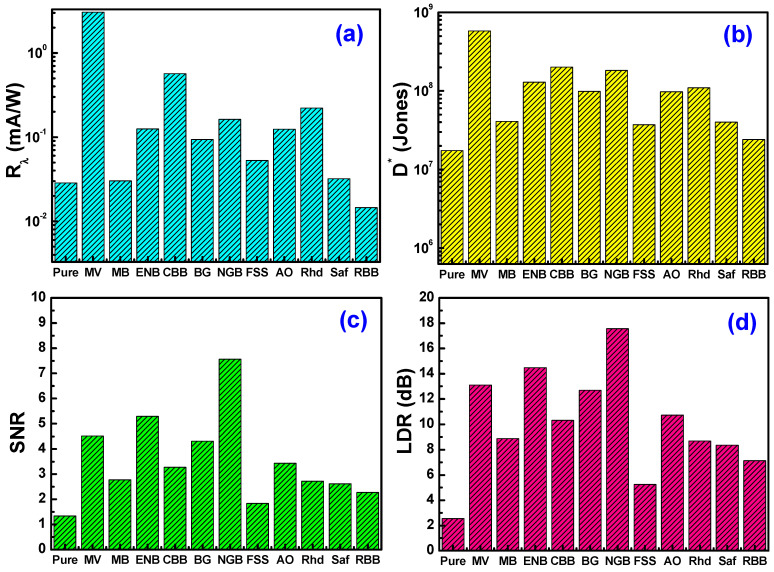
(**a**) Responsivity, (**b**) specific detectivity, (**c**) signal-to-noise ratio, and (**d**) linear dynamic range of Ag/chitosan/p-Si/Al photosensors.

In order to ensure the validity of the fabricated devices for photodetection applications, it was necessary to calculate some of the parameters of the photodetectors, such as their ability to sense weak optical signals (i.e., specific detectivity D*), their response to the light falling on them (i.e., responsivity *R*), strength of the response electrical signal relative to the noise signal (i.e., signal-to-noise ratio *SNR*), and the linearity of their performance to determine the extent of their ease of compatibility with the rest of the electronic components in the sensor circuits (i.e., linear dynamic range *LDR*). Considering the dominance of the shot noise mechanism in the noise current, the values of *R*, D*, *SNR*, and *LDR* were calculated as follows [34,38,41,46]:

(16) $$R=\frac{J_{illumination}-J_{dark}}{P_{in}},$$

(17) $$D^{*}=\frac{\sqrt{A\Delta f}}{NEP}=\frac{R}{\sqrt{2qJ_{dark}}},$$

(18) $$SNR=\left(\frac{J_{photo}}{J_{dark}}\right),$$

(19) $$LDR=20\log\left(\frac{J_{photo}}{J_{dark}}\right),$$

where Δ*f* is the signal bandwidth, *NEP* is the noise equivalent power, and *q* is the electronic charge. Figure 10 reveals the estimated values of *R*, D*, *SNR*, and *LDR* of the fabricated photosensors under 100 mW/cm^2^ illumination intensity and at −3 V bias voltage. Clearly, Ag/Cs-MV/p-Si photosensors showed a superior performance relative to the other fabricated photosensors with 3.05 mA/W, 5.79 × 10^8^ Jones, 4.5, and 13.1 dB for responsivity, detectivity, signal-to-noise ratio, and linear dynamic range, respectively. Table 4 shows a comparison between the estimated values of the photodetectors’ technical parameters relative to other organic/inorganic heterojunction photosensors. The prepared photosensors showed a remarkable improvement in the values of the sensing parameters compared to many previously prepared heterojunction sensors, and this is what may make the fabricated heterojunction during the study a strong nominee in the field of eco-friendly photosensors for wider industrialization. The differences between photoelectrical performance between the fabricated devices and other devices in the literature depend mainly on the device structure in terms of the work function of the utilized electrodes, area of the device, type of silicon substrate, doping concentration, and thickness of the spin-coated polymeric films. Furthermore, the interactivity between the utilized organic layer (pure and dyed chitosan film) and the silicon substrate’s surface will play a pivotal role in passivating the native surface defects and dangling bonds of the silicon surface to yield a high-quality junction of lower e-h recombinations and higher efficiency via controlling the interfacial dipole polarization.

## 4. Conclusions

In this study, thin films of pure chitosan and dyed ones with eleven different colored organic dyes were successfully prepared via the spin-coating method. Then, the molecular changes because of the pigmentation process were studied using FTIR, which showed the complete stability of the molecular structure of chitosan, noting the formation of electrostatic bonding and hydrogen bonding between the molecular structure of the dyes and the molecular structure of chitosan. The optical measurements of the prepared films showed a clear change in the extent of the absorbance and reflectivity of the prepared films with the change in the type of dye used, which led to a significant change in the gap energy values, where NGB-dyed chitosan films achieved the lowest value (2.88 eV) and Saf-dyed chitosan films achieved the lowest static refractive index (1.58). Regarding the nonlinear applicability, ENB-dyed chitosan films achieved the best performance with 1.21 × 10^−13^ esu and 2.65 × 10^−12^ e.s.u, for χ^(3)^, and n_2_, respectively. The photoluminescence and color parameters of all the fabricated films were investigated for evaluating their applicability for visible light filtering and shield applications, which were confirmed by the green-laser blocking experiment. On the scale of the laser cut-off performance, chitosan dyed with Rhd was the best. Eventually, the high absorbance of the prepared films in the UV and visible ranges was exploited in the manufacture of the heterojunction of these films as an absorbance enhancer with silicon for photodetection purposes. By analyzing the electrical results in the dark and light, many of the prepared junctions showed good photodetection behavior with a high response. In addition to that, some junctions experienced photovoltaic behaviors, which are suggested for solar cell applications, specifically chitosan films dyed with Saf and MV, which achieved the highest open-circuit voltage and short-circuit current density. The microelectronic parameters of the designed devices were evaluated, where the Ag/Cs-MV/p-Si device recorded a better performance relative to the other devices. Furthermore, the achieved responsivity and specific detectivity for Ag/Cs-MV/p-Si are considered high relative to many reported devices. Finally, these tunable, echo-friendly, easily processable films can be considered a powered commercialized platform for multifunctional optoelectronic and solar energy applications. This study needs more modifications to enhance the device’s performance, which may be induced by incorporating plasmonic nanoparticles to improve the light-harvesting capability and enhancing its conductivity, which will be performed in our future work. In addition, the dyeing level will be optimized and the influence of UV irradiation on the designed films will be studied for assessing the stability of the present films for window material applicability and usability for temperature shielding applications.

## Figures and Tables

**Table 1:** FTIR peak positions and molecular vibration assignment of the prepared pure and dyed chitosan thin films.

Assignment	Wavenumber (cm^−1^)											
	Pure	MV	MB	ENB	CBB	BG	NGB	FSS	AO	Rhd	Saf	RBB
	409	403	-	407	-	402	401	-	406	-	409	407
	418	418	414	421	426	416	421	410	426	419	429	417
	442	443	44	434	-	430	438	435	-	439	444	434
	454	-	456	453	455	451	449	459	457	-	459	445
	464	462	465	467	-	464	465	-	-	465	460	467
	-	-	-	-	480		479	472	-	-	-	-
	-	492	492	485	495	495	494	-	506	490	488	501
	525	522	527	513	512	518	517	519	521	524	520	523
	-	-	-	-	537	-	-	-	-	-	-	-
**δ (NH) + δ (C-O)**	559	559	556	553.69	560	558	554	566	558	558	554	559
	-	588	567	577	588	597	598	578	581	-	592	-
	605	604	603	605	604	607	617	604	605	600	608	607
	659	659	659	662	659	658	659	-	660	659	658	659
**δ (NH)**	707	704	706	710	690	701	607	710	698	-	709	705
	-	-	-	-	-	-	740	746	-	-	747	-
	-	801	-	810	808	809	801	805	-	-	-	800
**ν (C-N)**	895	895	894	890	895	895	895	895	894	895	892	894
	-	-	992	988	-	993	991	987	989	990	986	993
**ν (C-O)**	1027	1024	1027	1025	1028	1026	1027	1024	1025	1026	1024	1025
**δ (C-O) + ν (C-O)**	1063	1062	1064	1061	1065	1063	1066	1062	1063	1064	1061	1064
**νas (C-O-C)**	1151	1151	1151	1151	1152	1151	1150	1150	1151	1151	1151	1151
	-	-	-	-	-	1198	1199	-	1200	1198	1201	1198
**ν (NH_2_) + δ (OH)**	1261	1261	1262	1259	1258	1262	1261	1260	1259	1256	-	1261
**ν (C-N) amide III**	1326	1327	1327	1330	1327	1326	1322	1329	1325	1335	1322	1335
**δ(C=O) + τ (C-H) in CH3 group**	1376	1372	1378	1380	1375	1376	1376	1377	1376	1376	1376	1377
**δ(C-H) + ω(CH_2_)**	1419	1416	1418	1415	1420	1416	1409	1408	1415	1413	1415	1414
**ν (C=O) + ν (NH) amide II + δ (N-H) amine I**	1583	1584	1583	1596	1580	1582	1560	1580	1581	1587	1573	1580
**ν (C=O) + ν (C-O) + ν (NH) amide I**	1646	1645	1648	1651	1647	1646	1645	1645	1646	1635	1645	1649
**ν (C=O)**	-		-	-	-	-	-	1737	-	1754	-	-
**νas(C-H) of methylene**	2867	2865	2865	2865	2858	2867	2862	2870	2864	2867	2850	2863
**νs(C-H)**	2917	2919	2914	-	2919	2917	2914	2907	2914	2913	2918	2907
**ν(OH) + ν(NH)**	3292	3287	3290	-	3272	3289	3271	3284	3275	3294	3282	3285
	3356	3350	3355	3352	3347	3359	3359	3366	3357	3359	3341	3353

**Table 2:** Linear and nonlinear optical constants of pure and dyed chitosan thin films.

Optical Parameter		Pure	MV	MB	ENB	CBB	BG	NGB	FSS	AO	Rhd	Saf	RBB
Eg (eV)	Eg1	3.82	3.64	3.71	3.44	3.43	3.53	2.88	3.52	3.42	3.78	3.78	3.81
	Eg2	-	1.86	1.69	1.80	1.79	1.80	1.48	2.29	1.80	2.27	2.04	2.06
E_u_ (meV)		243	229	274	191	541	161	840	662	297	207	188	418
E_d_ (eV)		19.3	18.2	11.6	7.60	11.5	10.2	19.1	31.7	14.5	14.1	5.57	16.3
E_o_ (eV)		9.36	8.87	5.94	3.60	6.36	5.10	9.72	18.8	7.10	6.86	3.71	8.11
n_o_		1.75	1.75	1.72	1.76	1.68	1.73	1.72	1.64	1.74	1.75	1.58	1.73
ε_∞_		3.06	3.05	2.95	3.11	2.81	3.00	2.97	2.69	3.04	3.05	2.5	3.01
ε_L_		3.24	3.19	3.07	3.28	2.99	3.22	3.09	2.81	3.22	3.24	2.54	3.18
N/m* (×10^46^ g^−1^·cm^−3^)		6.05	5.07	4.55	6.25	5.49	6.52	5.48	4.33	6.25	6.50	0.86	5.67
ω_p_ (×10^14^ Hz)		2.32	2.14	2.07	2.35	2.31	2.42	2.26	2.11	2.37	2.41	0.99	2.27
ψ (eV)		0.152	0.141	0.136	0.154	0.152	0.159	0.148	0.139	0.156	0.158	0.065	0.149
E_P_ (eV)		0.094	0.098	0.097	0.106	0.113	0.112	0.105	0.107	0.109	0.100	0.053	0.105
E_F_ (eV)		0.024	0.022	0.021	0.024	0.024	0.025	0.023	0.021	0.025	0.025	0.008	0.023
χ^(1)^ (e.s.u)		0.164	0.163	0.163	0.168	0.144	0.159	0.156	0.134	0.163	0.164	0.119	0.160
χ^(3)^ (×10^−13^ e.s.u)		1.23	1.21	1.21	1.36	0.730	1.09	1.02	0.552	1.19	1.22	0.347	1.11
n_2_ (×10^−12^ e.s.u)		2.66	2.61	2.65	2.90	1.64	2.38	2.23	1.27	2.57	2.63	0.827	2.43

**Table 3:** Color parameters of pure and dyed chitosan thin films.

Color Parameter	Pure	MV	MB	ENB	CBB	BG	NGB	FSS	AO	Rhd	Saf	RBB
X	0.231	0.245	0.297	0.311	0.213	0.211	0.306	0.261	0.431	0.562	0.355	0.300
Y	0.21	0.188	0.277	0.299	0.201	0.234	0.300	0.569	0.449	0.385	0.322	0.286
L*	47.29	29.91	26.15	26.70	34.78	38.97	33.62	39.35	34.74	32.95	31.98	25.44
a*	−0.81	8.87	2.92	1.03	−1.39	−2.94	−9.39	−1.84	8.01	17.07	9.80	−0.58
b*	−1.34	−12.17	−9.54	−4.47	−6.45	−1.25	6.43	8.32	16.97	−7.85	3.30	−6.07
dE*	7.65	18.18	16.90	13.84	8.09	2.78	12.61	8.55	0.25	20.38	13.36	15.50
C*	1.57	15.06	9.98	4.59	6.59	3.19	11.38	8.52	18.76	18.79	10.34	6.10
h	238.77	306.08	286.99	282.95	257.85	202.94	145.60	102.47	64.74	335.32	18.62	264.58

L* lightness parameter, a* red-green axis, b* yellow-blue axis, dE* change in color, C* Chroma, h hue angle, X and Y are CIE coordinates.

**Table 4:** A comparative illustration between the figures of merit of the designed chitosan/p-Si heterojunction and other organic/Si heterojunctions for light sensing.

Device	Pin (mW/cm^2^)	R (mA/W)	D* (Jones)	SNR	LDR (dB)	Ref.
Ag/Cs/p-Si	100	0.029	1.74 × 10^7^	1.340	2.545	Present work
Ag/Cs-MV/p-Si		3.046	5.79 × 10^8^	4.517	13.09	
Ag/Cs-MB/p-Si		0.030	4.10 × 10^7^	2.778	8.875	
Ag/Cs-ENB/p-Si		0.125	1.29 × 10^8^	5.290	14.47	
Ag/Cs-CBB/p-Si		0.569	2.01 × 10^8^	3.27	10.30	
Ag/Cs-BG/p-Si		0.094	9.85 × 10^7^	4.308	12.69	
Ag/Cs-NGB/p-Si		0.163	1.82 × 10^8^	7.557	17.57	
Ag/Cs-FSS/p-Si		0.053	3.71 × 10^7^	1.830	5.251	
Ag/Cs-AO/p-Si		0.125	9.74 × 10^7^	3.434	10.72	
Ag/Cs-Rhd/p-Si		0.223	1.09 × 10^8^	2.718	8.686	
Ag/Cs-Saf/p-Si		0.032	4.01 × 10^7^	2.614	8.348	
Ag/Cs-RBB/p-Si		0.015	2.40 × 10^7^	2.269	7.118	
Al/PTCDI/p-Si	200	0.2	7.0 × 10^7^	-	-	[51]
Au/α-6 T/n-Si	2560	0.2	1.2 × 10^8^	57	35	[52]
Au/ABPQC/p-Si	100	6.5 × 10^−5^	1.25 × 10^6^	-	33.5	[53]
Co/MG/n-Si	400	0.08	-	-	-	[54]
Au/PaOEP/p-Si	80	8.5 × 10^−4^	1.2 × 10^3^	-	-	[55]
Au/MFCMP/p-Si	100	4.1 × 10^−5^	1.8 × 10^6^	-	-	[56]

## Data Availability

The data is available upon request from corresponding author.

## Supplementary Materials

The following supporting information can be downloaded at: https://www.mdpi.com/article/10.3390/ma16093475/s1. Figure S1: Schematic diagram of the designed MPS heterojunction. Figure S2: Tauc plot for energy gap estimation of the pure and dyed chitosan thin films. Figure S3: Urbach plot of the pure and dyed chitosan thin films. Figure S4: Dispersion behavior of pure and dyed chitosan thin films. Figure S5: *n*^2^ versus *λ*^2^ single oscillator model plot of the pure and dyed chitosan thin films. Figure S6: CIE 1931 chromatography of pure and dyed chitosan thin films. Figure S7: Spectral profile of the color strength of the pure and dyed chitosan thin films. Figure S8: (a) rectification ratio, (b) ideality factor, (c) reverse saturation current, (d) zero bias barrier height, (d) shunt resistance, and (d) series resistance of Ag/chitosan/p-Si/Al structured devices under dark conditions. Figure S9: Interface states density profile of the designed devices. Figure S10: Reverse photocurrent density under 100 mW/cm^2^ illumination of Ag/chitosan/p-Si/Al structured devices. Figure S11: Open circuit voltage and short circuit current density of Ag/chitosan/p-Si/Al photosensors. Table S1: Chemical information about the utilized organic dyes. Table S2: Viscosity coefficient of pure and dyed chitosan solutions. Table S3: Thickness of the prepared pure and dyed chitosan thin films.
