# Supplementary material for: Fabrication and Characterization of Eco-Friendly Thin Films as Potential Optical Absorbers for Efficient Multi-Functional Opto-(Electronic) and Solar Cell Applications
Source: Materials (Basel). 2023 Apr 29;16(9):3475. doi: 10.3390/ma16093475 (PMC10180174; doi:10.3390/ma16093475)
Supplement: Supplementary file 1 [file materials-16-03475-s001.zip › materials-2320452-supplementary.pdf]

Supporting material

Fabrication of Eco-Friendly Thin Films as Potential Optical Absorber for Efficient Multi-functional Opto-(electronic) and Solar Energy Applications

Mohamed H. El-Newehy [1,2] *, Ahmed M. El-Mahalawy[3], Badr M. Thamer[1,] and Meera Moydeen Abdul Hameed[1]

1 Department of Chemistry, College of Science, King Saud University, Riyadh 11451, Saudi Arabia
2 Department of Chemistry, Faculty of Science, Tanta University, Tanta 31527, Egypt.
3 Thin Films Laboratory, Physics Department, Faculty of Science, Suez Canal University, 41522, Ismailia, Egypt.
* Correspondence: melnewehy@ksu.edu.sa

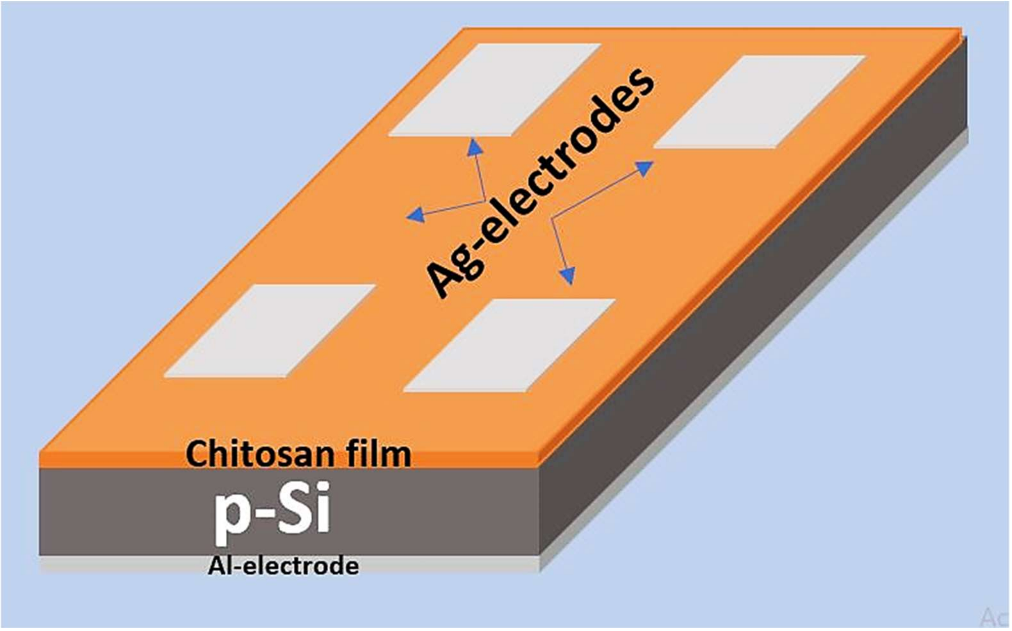

Fig.S1: Schematic diagram of the designed MPS heterojunction.

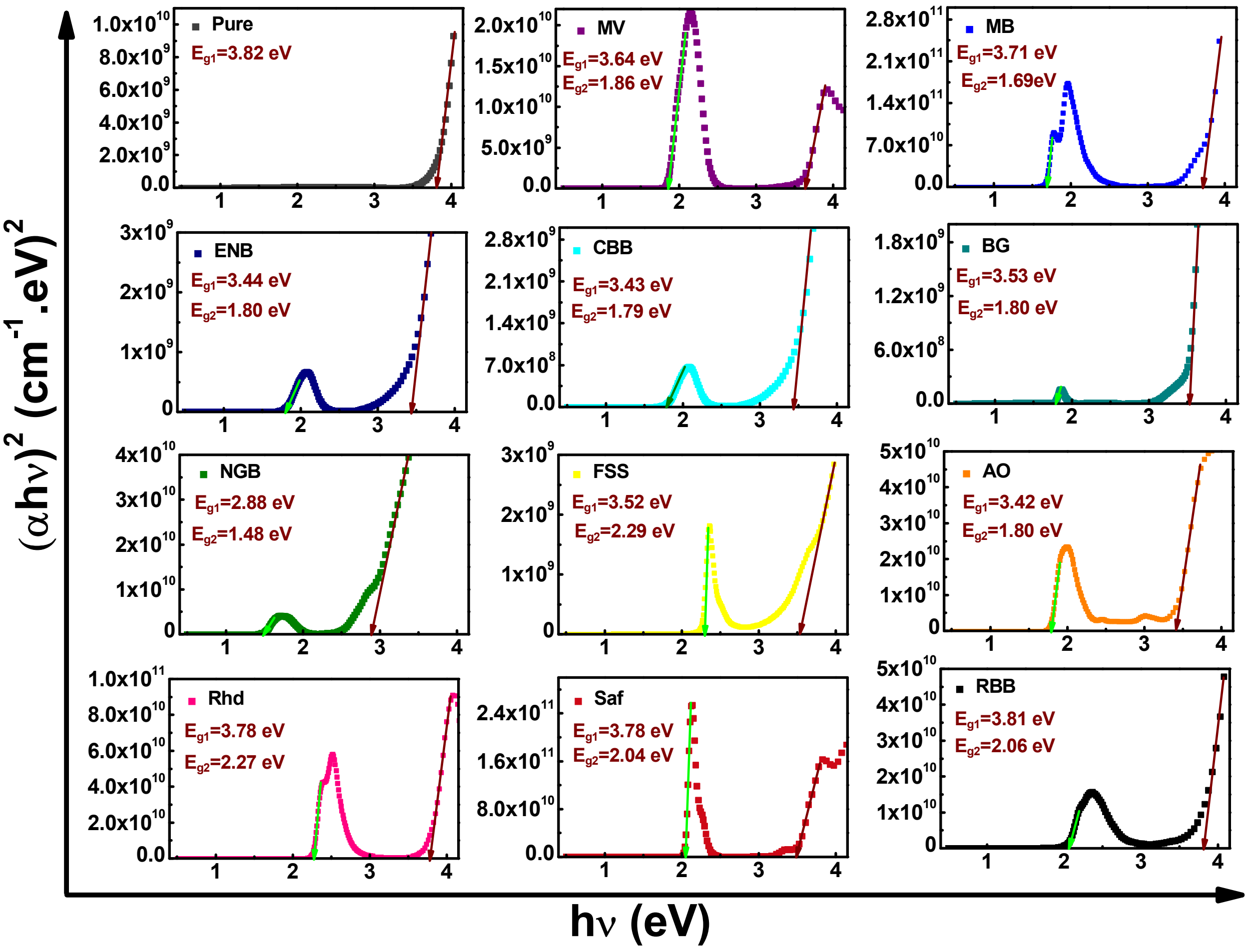

Fig.S2: Tauc plot for energy gap estimation of the pure and dyed chitosan thin films.

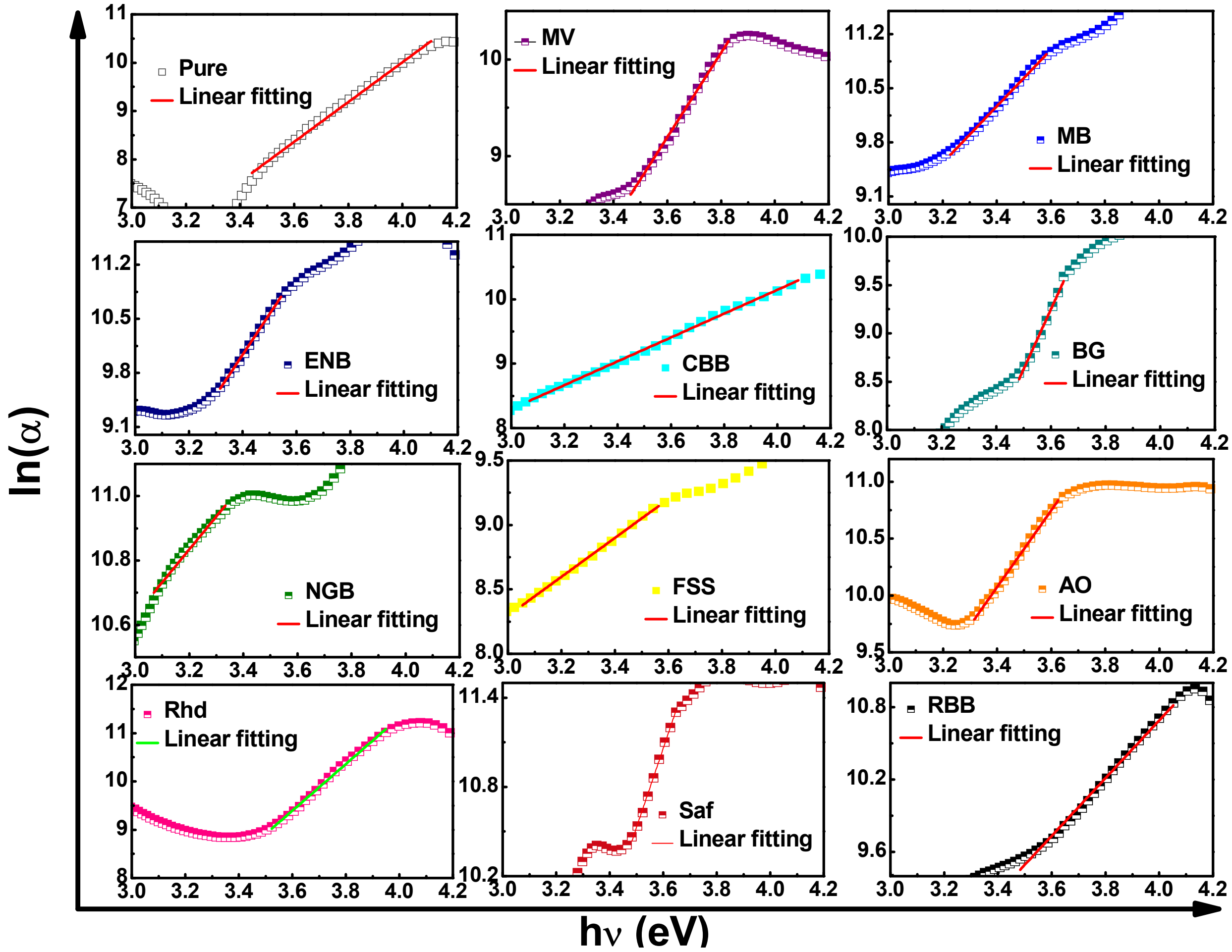

Fig.S3: Urbach plot of the pure and dyed chitosan thin films.

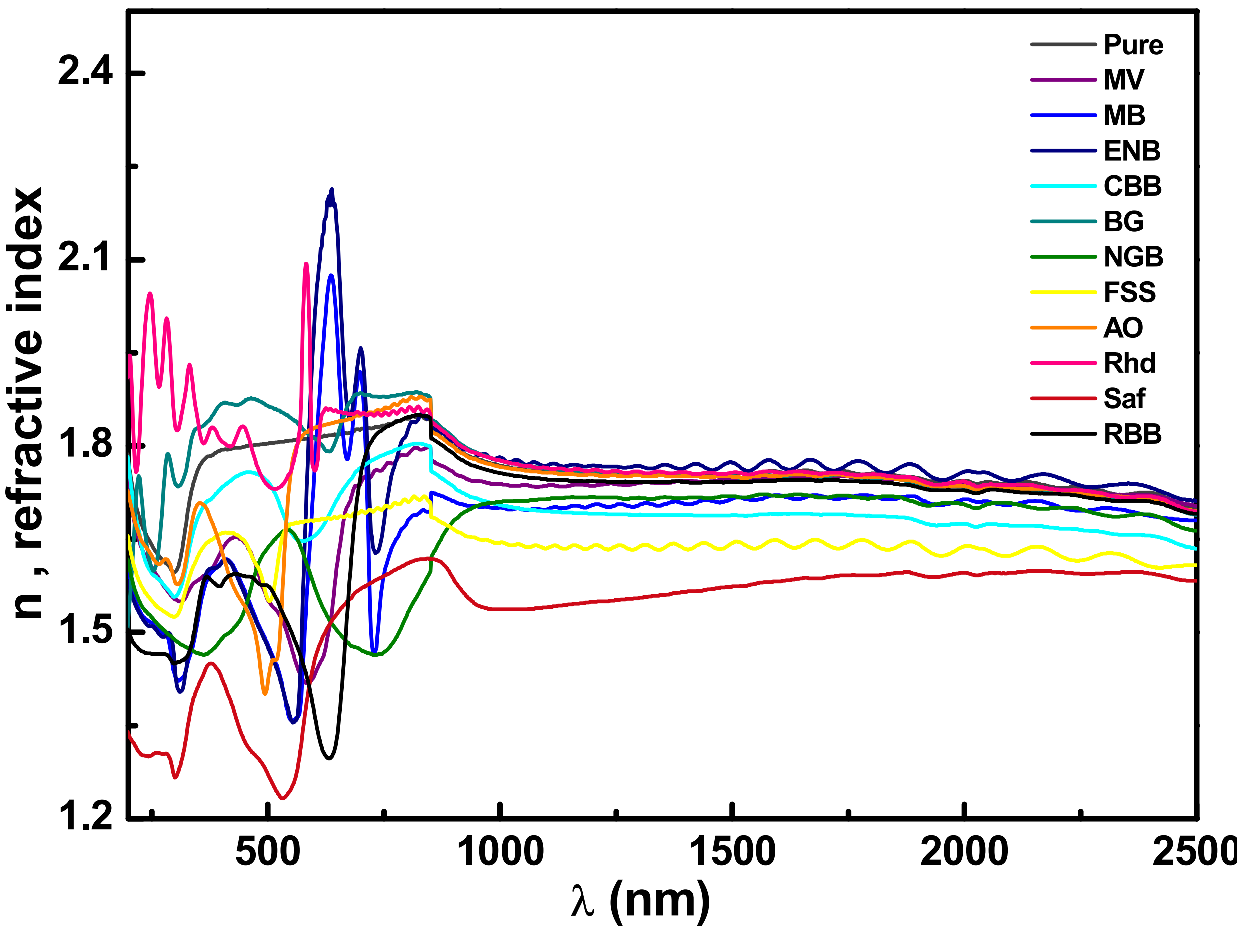

Fig.S4: Dispersion behavior of pure and dyed chitosan thin films.

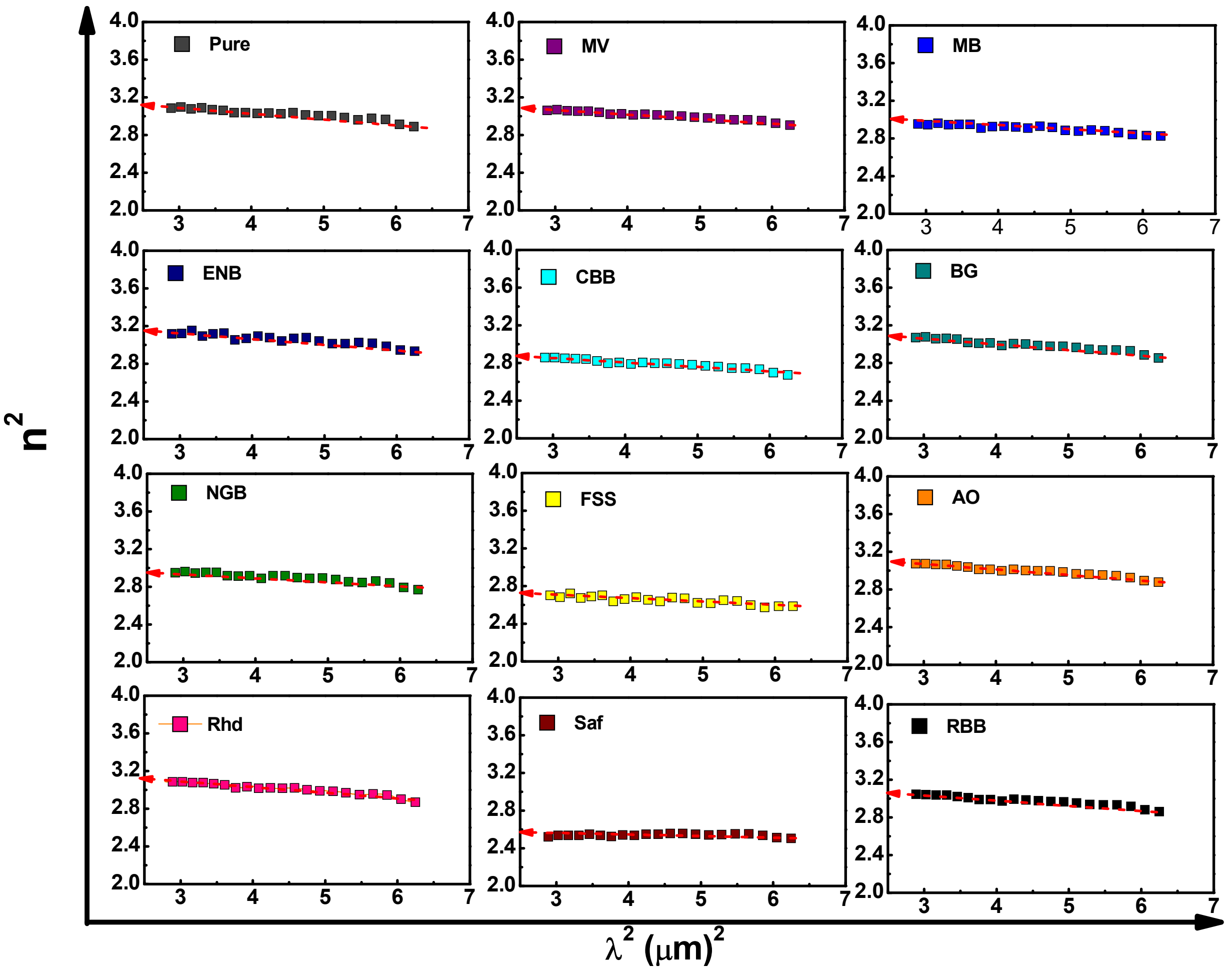

Fig.S5: n^2 versus λ^2 single oscillator model plot of the pure and dyed chitosan thin films.

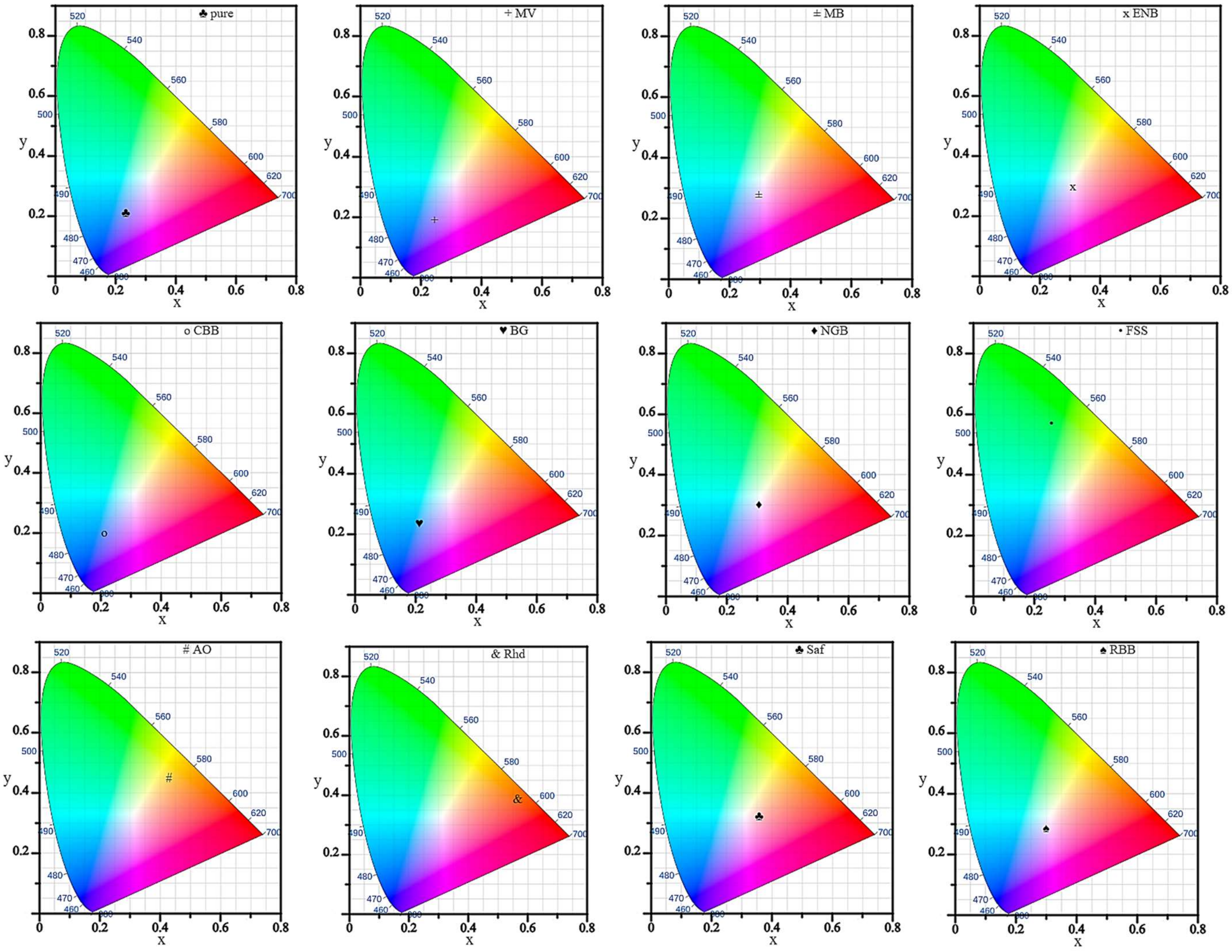

Fig.S6: CIE 1931 chromatography of pure and dyed chitosan thin films.

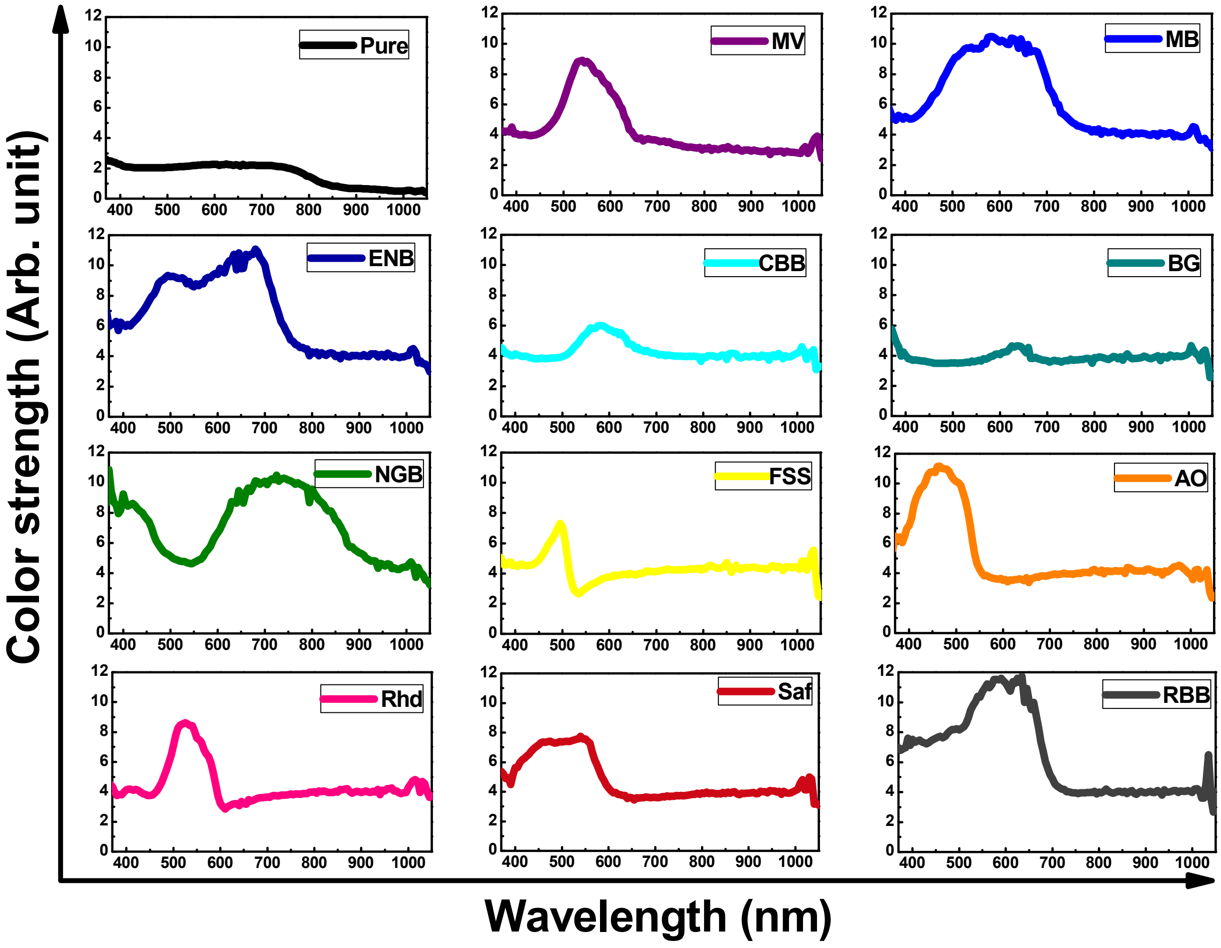

Fig.S7: Spectral profile of the color strength of the pure and dyed chitosan thin films.

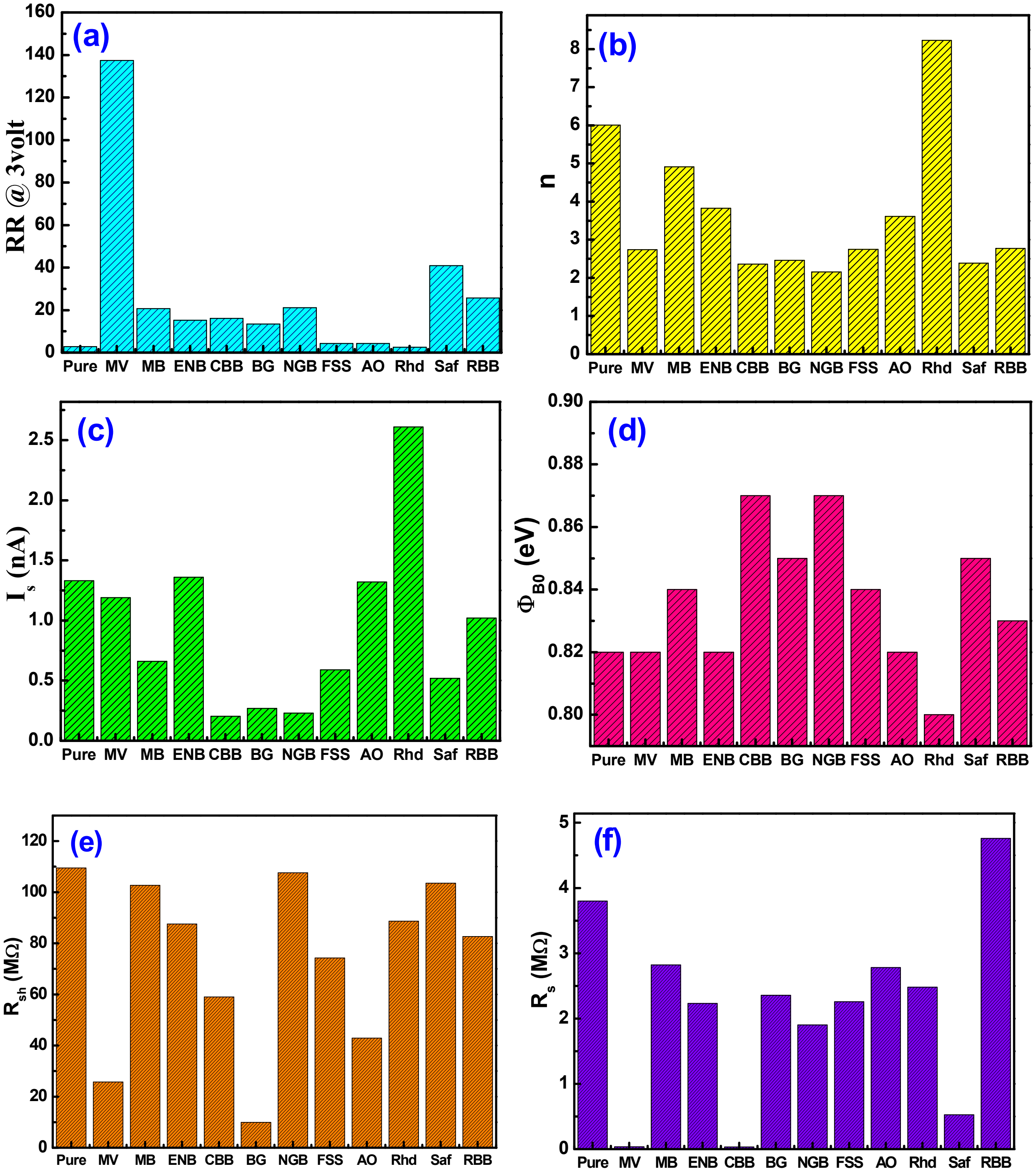

Fig.S8: (a) rectification ratio, (b) ideality factor, (c) reverse saturation current, (d) zero bias barrier height, (d) shunt resistance, and (d) series resistance of Ag/chitosan/p-Si/Al structured devices under dark conditions.

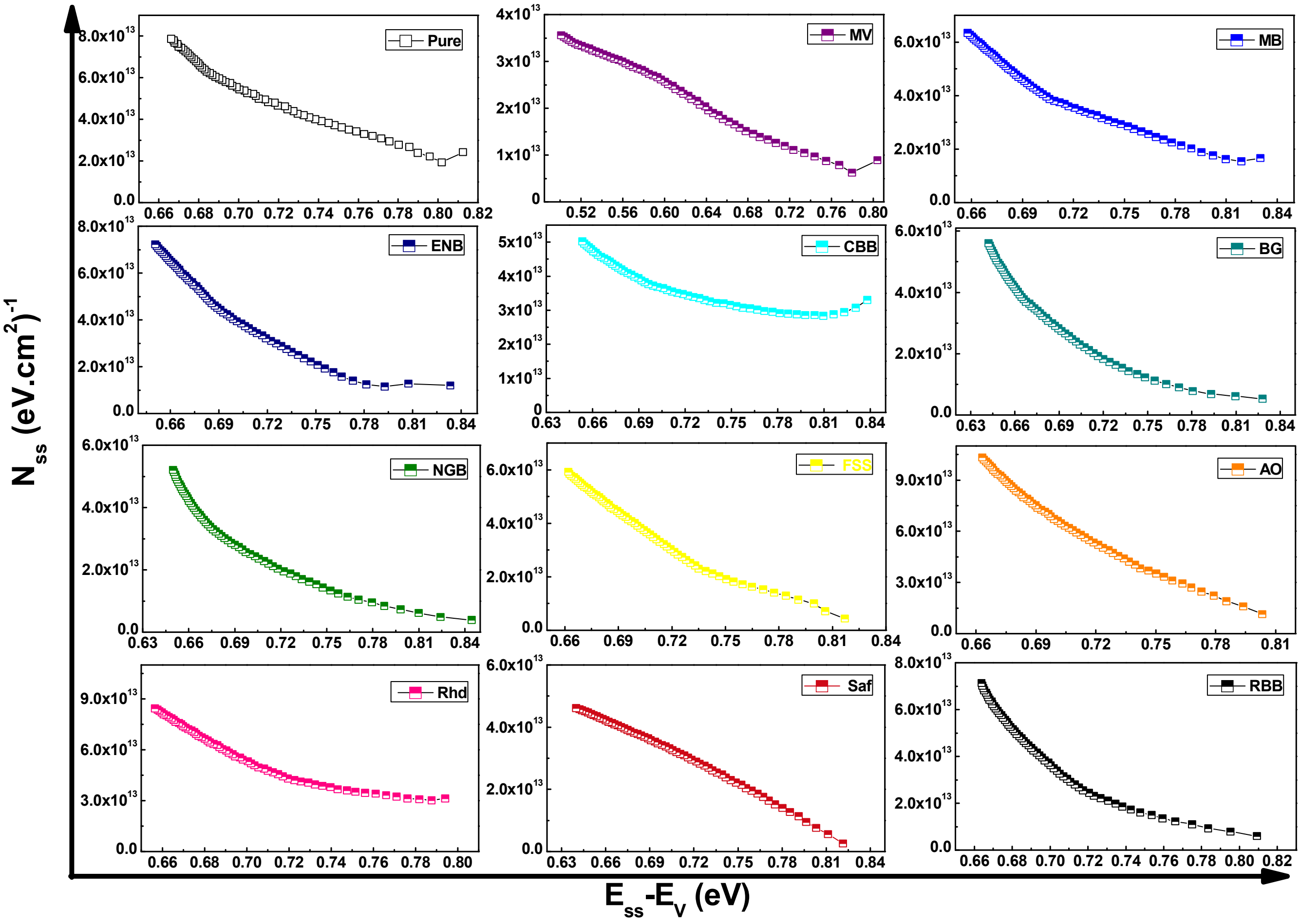

Fig.S9: Interface states density profile of the designed devices.

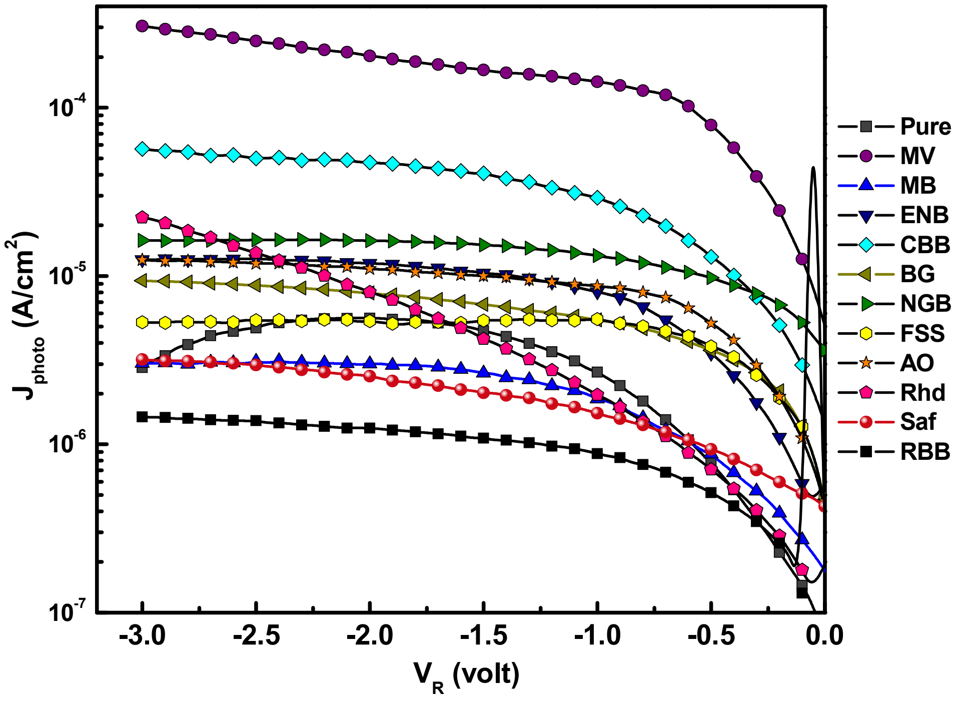

Fig.S10: Reverse photocurrent density under 100 mW/cm^2 illumination of Ag/chitosan/p-Si/Al structured devices.

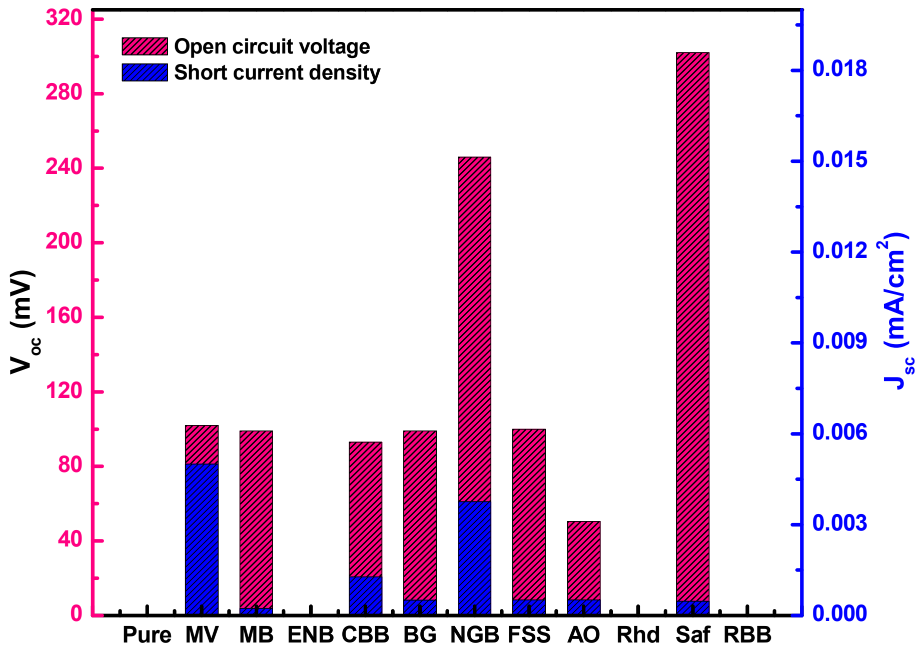

Fig.S11: Open circuit voltage and short circuit current density of Ag/chitosan/p-Si/Al photosensors.

Table.S1: Chemical information about the utilized organic dyes.

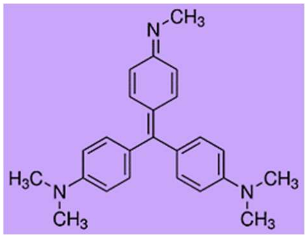<br>Name: Methyl Violet 6B<br>Symbol: MV<br>Molecular formula: $C_{24}H_{28}N_3Cl$<br>Molecular weight (g/mol): 379.9	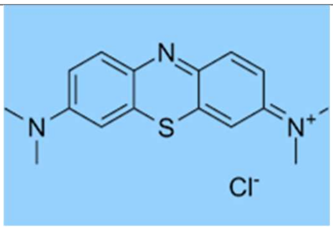<br>Name: Methylene Blue<br>Symbol: MB<br>Molecular formula: $C_{16}H_{18}ClN_3S$<br>Molecular weight (g/mol): 319.85	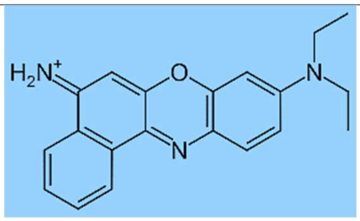<br>Name: Ethyl Nile blue<br>Symbol: ENB<br>Molecular formula: $2(C_{20}H_{20}N_3O)$ SO_4<br>Molecular weight (g/mol): 732.85
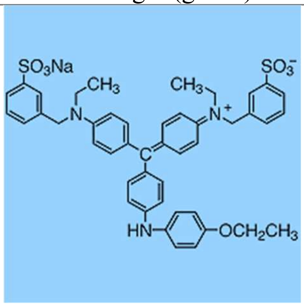<br>Name: Coomassie Brilliant Blue R<br>Symbol: CBB<br>Molecular formula:<br>$C_{45}H_{44}N_3NaO_7S_2$<br>Molecular weight (g/mol): 825.97	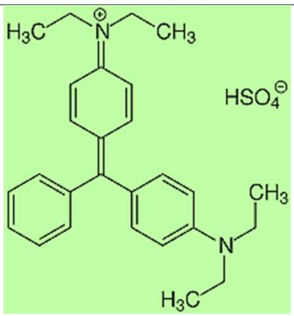<br>Name: Brilliant Green<br>Symbol: BG<br>Molecular formula: $C_{27}H_{34}N_2O_4S$<br>Molecular weight (g/mol): 482.63	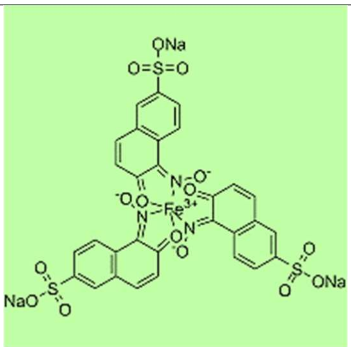<br>Name: Naphthol Green B<br>Symbol: NGB<br>Molecular formula:<br>$C_{30}H_{15}FeN_3Na_3O_{15}S_3$<br>Molecular weight (g/mol): 878.46
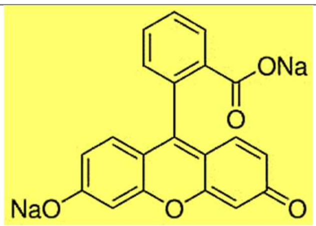<br>Name: Fluorescent Sodium Salt<br>Symbol: FSS<br>Molecular formula: $C_{20}H_{10}Na_2O_5$<br>Molecular weight (g/mol): 376.27	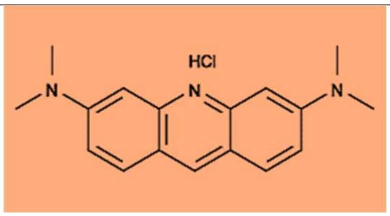<br>Name: Acridine Orange<br>Symbol: AO<br>Molecular formula: $C_{17}H_{19}N_3$<br>Molecular weight (g/mol): 265.36	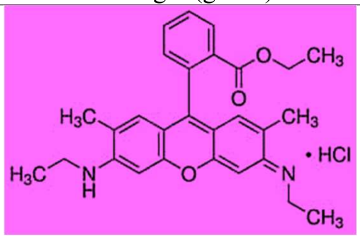<br>Name: Rhodamine 6G<br>Symbol: Rhd<br>Molecular formula: $C_{28}H_{31}N_2O_3Cl$<br>Molecular weight (g/mol): 479.01
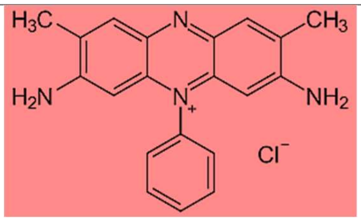<br>Name: Safranine O<br>Symbol: Saf<br>Molecular formula: $C_{20}H_{19}N_4Cl$<br>Molecular weight (g/mol): 350.84	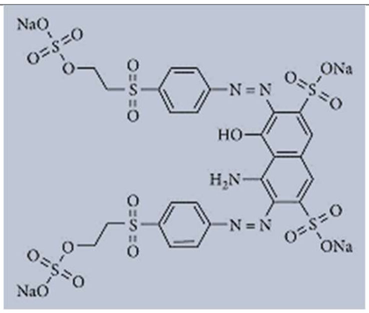<br>Name: Remazol Black B<br>Symbol: RBB<br>Molecular formula: $C_{26}H_{21}N_5Na_4O_{19}S_6$<br>Molecular weight (g/mol): 991.82	

Table.S2: Viscosity coefficient of pure and dyed chitosan solutions.

Solution	Viscosity (Pa. s)	Solution	Viscosity (Pa.s)
Pure chitosan	5.72 ± 0.083	**Chitosan + NGB**	2.98 ± 0.125
Chitosan + MV	3.81 ± 0.065	**Chitosan + FSS**	3.62 ± 0.032
Chitosan + MB	3.78 ± 0.127	**Chitosan + AO**	2.89 ± 0.098
Chitosan + ENB	3.65 ± 0.207	**Chitosan + Rhd**	3.21 ± 0.079
Chitosan + CBB	2.67 ± 0.122	**Chitosan + Saf**	3.69 ± 0.011
Chitosan + BG	3.11 ± 0.062	**Chitosan + RBB**	3.91 ± 0.065

Table.S3: Thickness of the prepared pure and dyed chitosan thin films.

Film	Thickness (nm)	Film	Thickness (nm)
**Pure**	394	**NGB**	400
**MV**	399	**FSS**	400
**MB**	401	**AO**	395
**ENB**	395	**Rhd**	350
**CBB**	400	**Saf**	403
**BG**	398	**RBB**	390
